# CuO/PMMA Polymer Nanocomposites as Novel Resist Materials for E-Beam Lithography

**DOI:** 10.3390/nano11030762

**Published:** 2021-03-17

**Authors:** Georgia Geka, George Papageorgiou, Margarita Chatzichristidi, Andreas Germanos Karydas, Vassilis Psycharis, Eleni Makarona

**Affiliations:** 1 Department of Chemistry, National and Kapodistrian University of Athens, Zografou, 157 71 Athens, Greece; georgia.geka101@gmail.com; 2 Institute of Nanoscience and Nanotechnology, NCSR “Demokritos”, Aghia Paraskevi, 153 10 Athens, Greece; g.papageorgiou@inn.demokritos.gr (G.P.); v.psycharis@inn.demokritos.gr (V.P.); 3 Institute of Nuclear and Particle Physics, NCSR “Demokritos”, 153 10 Athens, Greece; karydas@inp.demokritos.gr

**Keywords:** polymer nanocomposites, CuO nanostructures, PMMA, e-beam lithography, resist process engineering, X-ray fluorescence, chemical synthesis

## Abstract

Polymer nanocomposites have emerged as a new powerful class of materials because of their versatility, adaptability and wide applicability to a variety of fields. In this work, a facile and cost-effective method to develop poly(methyl methacrylate) (PMMA)-based polymer nanocomposites with copper oxide (CuO) nanofillers is presented. The study concentrates on finding an appropriate methodology to realize CuO/PMMA nanocomposites that could be used as resist materials for e-beam lithography (EBL) with the intention of being integrated into nanodevices. The CuO nanofillers were synthesized via a low-cost chemical synthesis, while several loadings, spin coating conditions and two solvents (acetone and methyl ethyl ketone) were explored and assessed with regards to their effect on producing CuO/PMMA nanocomposites. The nanocomposite films were patterned with EBL and contrast curve data and resolution analysis were used to evaluate their performance and suitability as a resist material. Micro-X-ray fluorescence spectroscopy (μ-XRF) complemented with XRF measurements via a handheld instrument (hh-XRF) was additionally employed as an alternative rapid and non-destructive technique in order to investigate the uniform dispersion of the nanofillers within the polymer matrix and to assist in the selection of the optimum preparation conditions. This study revealed that it is possible to produce low-cost CuO/PMMA nanocomposites as a novel resist material without resorting to complicated preparation techniques.

## 1. Introduction

Polymer nanocomposites, defined as polymers containing fillers with at least one dimension smaller than 100 nm at very low loadings (<5 vol. %), have emerged as a new and promising class of materials for a wide range of applications, which may span from the automotive [1] to the textile industry [2] or even bioengineering [3]. More importantly, polymer nanocomposites can be used as alternative building blocks or as the functional core of novel micro/nano-electronic devices [4,5,6,7,8]. This broadening of applicability is due to their very nature. In contrast to traditional polymer composites with high loadings (>50% *w*/*v*) of micrometer-size fillers, which have been used for almost 100 years [9], the recent progress of nanotechnology has provided a plethora of nanofillers, which, even at low % vol. loadings can drastically enhance and modify the polymer’s properties with respect to its bulk counterpart [10]. Hence, research efforts into polymer nanocomposites has revolved around the successful incorporation of nano-sized fillers into polymers so as to take full advantage of the nanofillers’ multi-faceted nature and to develop a new class of organic/inorganic materials of enhanced properties and multi-functionality [11,12]. Of particular interest is the fact that the newly-developed properties of the nanocomposites are largely different due to the actual morphology of the selected nanofillers and strongly depend on whether the nanofillers are two-dimensional (2D) layered structures, one-dimensional (1D) fibrous or zero-dimensional (0D) spherical ones [10]. Another critical parameter when creating polymer nanocomposites is the uniform dispersion of these isotropic or anisotropic nano-sized fillers, since the distribution itself controls the ultra-large interfacial area per unit volume between nano-scale fillers and host polymers, which in itself dictates the composite’s properties [10].

However, a major hurdle in the development of polymer nanocomposites, and in particular of nanocomposites of inorganic fillers, such as metal oxide nanoparticles, is the difficulty in obtaining homogeneous dispersions within the polymer matrix and in preventing agglomeration/aggregation and sedimentation of the nanofillers [13,14,15]. This difficulty mainly arises from two facts: (1) nanofillers are typically hydrophilic, while polymers are typically hydrophobic, and (2) nanofillers being very small in size (<100 nm) tend to agglomerate in order to minimize the surface to volume ratio, and in turn, the surface free energy of the system. The driving force for the agglomeration process is the van der Waals attraction. In general, the agglomerates are hard to break and do not produce the intended properties’ enhancement, even when dispersed in the polymer matrix in a homogeneous manner.

This work has concentrated on the development of a relatively facile approach for the creation of CuO/Poly(methyl methacrylate) (PMMA) nanocomposites as a novel resist material for electron beam lithography (EBL) with the final aim of being employed as the functional material in nanoelectronic devices, such as gas sensors. In most reported works, such as the those of the Gonsalves group since the early 2000s [16,17,18], the incorporation of nanofillers of sizes less than 10 nm inside resist hosts is performed with the aim of improving the resolution without sacrificing the inherent sensitivity and contrast. However, the incentive of the present work is to add functionalities to the resist by the inclusion of CuO nanostructures with typical sizes of 10–30 nm and to explore whether the composite PMMA can still be patterned by EBL and to what extent its properties (resolution, sensitivity) may be affected.

While grafting has been the most common method to enhance the nanofiller miscibility (e.g., References [19,20]), the suggested approach in this work entails the use of polar solvents and physical mixing so as to keep the cost and preparation time as low as possible, and it was based on the reported results of Botsi et al. [21]. Physical mixing of CuO nanofillers with the polymer host has also been reported in other works [22,23,24], but in all cases, the films were merely formed by drop-casting and lithographic patterning was not attempted. CuO was selected as the nanofiller as it has been proven to be one of the most promising and versatile metal oxides exhibiting a remarkable spectrum of properties, such as catalytic activity [25], energy storage capabilities [26], optoelectronic [27,28] and antibacterial properties [29], and most notably gas sensing properties [30,31,32]. This work was mainly devised with the latter in mind so as to create a novel CuO/PMMA resist material readily applicable for the development of polymer-based novel gas sensors [24,33]. PMMA was chosen as the polymer host, since it is one of the most widely employed resists in EBL donned with optical transparency in the UV-VIS part of the spectrum, good mechanical properties and chemical stability.

The present study had, as a first step, the synthesis of appropriate CuO nanofillers. Subsequently, several CuO/PMMA polymer nanocomposite solutions were prepared and tested as positive tone EBL resists over silicon substrates. Contrast curve data and resolution analysis of the patterned CuO/PMMA films were used to evaluate the performance of the polymer nanocomposites as resist materials and the suitability of the suggested methodology as an alternative cost-efficient method for the production of CuO/PMMA nanocomposites. Within the framework of this study, four critical parameters were explored: the loading of the PMMA matrix with CuO nanofillers, the substrate coating conditions, the stability of the nanocomposite solutions over time and the role of the solvent comparing acetone to methyl ethyl ketone (MEK). The studies were complemented by Micro-X-ray fluorescence spectroscopy (μ-XRF) and a handheld XRF (hh-XRF), which were employed, to the best of our knowledge, for the first time to investigate the miscibility of the nanofillers within the polymer matrix and to assist in the selection of the optimum preparation conditions.

## 2. Materials and Methods

The study was conducted in two phases.

Phase 1 concentrated on synthesizing the appropriate CuO nanofillers and verifying the proof-of-concept of the suggested methodology for the production of CuO/PMMA polymer nanocomposites as EBL resists. A low-cost solution-based method was employed for the synthesis of the CuO nanofillers, because of its cost-efficiency and nanoparticle design versatility through simple key parameters, such as the temperature and the precursor concentration. After the synthesis of the appropriate nanofillers, three parameters were studied: the loading of the PMMA matrix with CuO nanofillers, the substrate coating conditions and the stability of the nanocomposite solutions over time.

Phase 2 focused on the role of the solvent; methyl ethyl ketone (MEK) was compared to acetone in terms of the suitability and performance of the CuO/PMMA nanocomposites as EBL resists.

### 2.1. CuO Nanofiller Synthesis

**Figure 1:**
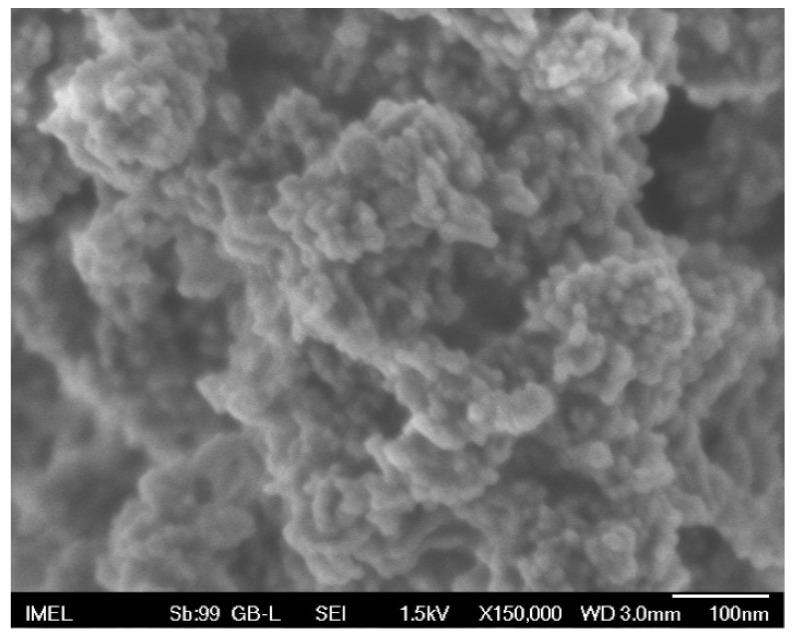
SEM image of the CuO nanopowder selected to be used as the nanofiller for the PMMA composites. Magnification: ×150,000; Scale bar: 100 nm.

The CuO nanofillers were synthesized following a low-cost, wet chemical method, according to which copper acetate was hydrolyzed by sodium hydroxide (NaOH) in an aqueous solution followed by thermal decomposition. The chosen method was in essence a variation of the reduction of copper acetate with NaOH, as reported by Gupta et al. [34]. In brief, copper (II) acetate monohydrate (Sigma Aldrich/Merck KGaA, Darmstadt, Germany) was dissolved in DI water at room temperature to form a 65 mM solution. The solution was then placed on a hot plate and was heated under continuous magnetic stirring up to 80 °C. At that point, a 500 mM NaOH aqueous solution was added drop-wise in 2 mL doses until a copper acetate to NaOH molar ratio of 1:4 was reached. Upon addition of NaOH the translucent blue solution turned gradually opaque blue (Figure S1, Supplementary Materials, SM). The final solution was left under constant stirring at 80 °C for 2 h, during which a black sediment, characteristic of CuO synthesis, was formed and the solution turned transparent (Figure S1). The solution was left undisturbed to cool to room temperature overnight. Finally, the black precipitate was centrifuged (Kubota 2420, Kubota Corporation Tokyo, Japan) and washed with distilled water 3 times, and dried at 60 °C for 20 h and then at 90 °C for 24 h in an oven in presence of atmospheric air (Figure S1). The specific parameters of the synthesis (concentration, copper salt-to-NaOH molar ratio, temperature, duration of synthesis, etc.) were chosen after various combinations had been tested (Table S1). The final selection of the synthesis parameters was based on the requirement that they should lead to the formation of well-defined nanostructures of uniform average size (see Section 1 of SM for details, Figures S2 and S3 and Table S1). The nanopowder chosen for the production of the nanocomposites is shown in Figure 1. It consisted of almost spherical nanoparticles of pure CuO with an average diameter of ~10 nm.

### 2.2. CuO Nanofiller Characterization

The CuO powders were structurally and morphologically characterized by Field-emission Scanning electron Microscopy (FE-SEM) with a JEOL JSM-7401f (Tokyo, Japan) and X-ray Diffraction (XRD) with q D500 SIEMENS Bragg-Brentano diffractometer, equipped with a pyrolytic graphite monochromator at a diffracted beam position and using Cu Kα radiation (CuKα1 Å: 1.54060, CuKa2 Å: 1.54439). The power conditions were set at 40 kV/35 mA, in addition to the aperture and the anti-scatter slit, which were set at 1°. The continuous step-scanning technique was used at steps of 0.03° with a measuring time of 2 s/step and the recorded 2θ range was from 2.0° to 100.0°. The XRD results are summarized in Figure S2.

### 2.3. PMMA Preparation

5% *w*/*w* PMMA in propylene glycol monomethyl ether acetate (PGMEA) solutions were prepared for Phase 1 and 6% *w*/*w* in PGMEA were prepared for Phase 2 using PMMA with a molecular weight MW = 996 k from Sigma Aldrich. Dissolution of PMMA was aided by the use of a magnetic stirrer in conjunction with low thermal plate heating (<70 °C) for 72 h.

### 2.4. PMMA/CuO Polymer Nanocomposite Solutions

In order to form the polymer nanocomposites, the following method was used in both phases: CuO nanopowder was added to a pre-calculated volume of acetone and the solution was vigorously stirred on a magnetic stirrer for 30 min at 40 °C in an effort to break up as many agglomerates as possible. A specified amount of acetone was added into the prepared PMMA/PGMEA solution prior to the addition of the nanofillers and the new solution was stirred at 40 °C for 30 min. Subsequently, the two mixtures were combined, so that after the addition of the acetone-CuO solution, a 4% *w*/*v* PMMA solution was obtained, while the loading of CuO nanofillers was 1% *w*/*v*, 2% *w*/*v* or 3% *w*/*v*. The final CuO/PMMA solution was stirred for another 30 min at 40 °C to improve homogenization (Figure S5a). Additional CuO/PMMA solutions were prepared, in which a small amount of deflocculant was added (Darvan C, Vanderbilt Minerals, LLC, Norwalk, CT, USA) to study its effect on the stability and homogenization of the polymer nanocomposite solutions. Darvan C is an ammonium salt of poly (methacrylic acid) with an average molecular weight of 10,000–16,000 g/mol and is commercially available as an aqueous solution with an active content 25%. Finally, a 4% *w*/*v* PMMA-acetone solution without any nanofillers was prepared to be used as a reference, hereafter referred to as REF. In Phase 2, the same preparation procedure was followed with the concentration of the nanofillers kept fixed at 1% *w*/*v*. Two different solvents were studied, acetone and methyl ethyl ketone (MEK), so as to evaluate the role of the solvent in the nanocomposite preparation. Three different solutions were prepared for each solvent; one containing no nanofillers used as a reference, one with only CuO nanofillers and one with CuO nanofillers and a small amount of deflocculant (Darvan C).

### 2.5. Electron Beam Lithography

The CuO/PMMA nanocomposites were tested as positive tone EBL resists according to the following procedure. All CuO/PMMA solutions were spin-coated onto 2.5 × 2.5 cm^2^ Si pieces obtained after dicing 3” Si wafers. The silicon substrates were thoroughly cleaned prior to the deposition with organic solvents and a piranha solution. Three different rotation speeds were tested during Phase 1, namely 1000 rpm, 3000 rpm and 4000 rpm (30 s) in order to select the most appropriate spin-coating conditions. Three different nanofiller loadings were tested, namely 1%, 2% and 3% *w*/*v*. Additionally, the addition of deflocculant was examined. All the samples underwent a post apply bake (PAB) at 180 °C for 1 min. For Phase 1, the coated samples were named after the CuO/PMMA solution used as follows: “X%CuO-Yk”, where X was the concentration of CuO (1%, 2% or 3% *w*/*v*) and Y was the spin coating speed (1k, 3k or 4k corresponding to 1000 rpm, 3000 rpm and 4000 rpm, respectively).“REF-Yk” corresponds to the reference samples prepared by the reference 4% *w*/*v* PMMA-acetone solution without any nanofillers spin-coated at Yk rpm. In the case of the deflocculant addition, the sample name contains the ending “-DF”. All samples that were studied in Phase 1 are listed in Table 1. The reported thicknesses of the films were determined by stylus profilometry (Ambios XP2, Ambios Technology, Inc, Milpitas, CA, USA) after development.

During Phase 2, the spin coating speed was fixed to 1000 rpm and the CuO loading to 1% *w*/*v*, since the main goal was to examine the effect of the solvent. Two series of samples were prepared, one for acetone and one for MEK. Each series contained one reference film produced with the “bare” PMMA solution, one film created with the 1% CuO/PMMA solution, one with the 1% CuO/PMMA solution containing the deflocculant and one with the 1% CuO/PMMA solution without deflocculant being filtered during the drop-casting using PTFE filters with 0.2 μm pores (Machery-Nagel GmbH & Co, Dueren, Germany). The thickness of the films was determined both via ellipsometry (M2000-F, J.A. Woollam Co., Lincoln, NE, USA) after PAB and via stylus profilometry after development.

For Phase 2, the samples were named as follows “X-REF”, “X-CuO”, “X-DF” and “X-FIL”, where X denotes the solvent used (X: ACE for acetone; MEK for MEK), “REF” corresponds to the reference solutions without nanofiller, “CuO” denotes that only nanofillers were added, “DF” denotes the addition of deflocculant and “FIL” denotes the filtering procedure during drop-casting. The samples of Phase 2 are summarized in Table 2.

For all samples in both phases, contrast curve patterning was conducted using a Raith EBPG5000+ e-beam writer (Raith GmbH, Dortmund, Germany) operating at 100 keV, in order to compare and characterize resist formulations via their response to exposure dose. Microscale structures (200 μm-wide squares, Figure S4a) intended for contrast curve data acquisition were exposed without the proximity effect correction at a detailed set of exposure doses (50–645 µC/cm^2^), with a 15 µC/cm^2^ step. Resolution patterns were exposed at a range of base doses (330–700 µC/cm^2^), below and above the observed dose to clear, with a 40 µC/cm^2^ dose step. 200 µm-long rectangular ribbons of variable width (300 nm, 500 nm, 1 µm, 5 µm, 10 µm, and 20 µm) were defined both by direct exposure (grooves) and by exposure of their periphery (protruding ridges), in order to probe the ability to design and transfer patterns onto the nanocomposite/resist films under different exposure conditions destined for different applications and architectures (see the schematic representation Figure S4b,c). Patterns for resolution studies were designed on KLayout and lithographic data preparation, including proximity effect correction, was performed using Beamer from GenISys. EBL was conducted using a 30 nA e-beam current and beam shot pitch was set to 25 nm. The development duration was set to 60 s. A 7:3 isopropanol/DI water co-developer solution was used for the development of samples followed by isopropanol rinse and N_2_ blow.

Contrast curve data (remaining film thickness in the exposed area) were acquired via stylus profilometry. Contrast curves measure the resist formulations’ sigmoidal response to exposure dose, while contrast (γ) is a dimensionless parameter that measures the films’ characteristic ability to conform to dose variations, under particular processing conditions. Typically, γ is extracted from the linear portion of the curve close to zero thickness, however, in our analysis, calculation of γ values is based on best curve fit, using the Ziger-Mack methodology [35]. The resist formulations’ performance, in terms of resolution, was assessed via optical microscopy and qualitative inspection of e-beam defined resolution patterns. Structural response to exposure dose bears information on the limitations and capabilities of resist variations and is discussed in the Results section.

### 2.6. Micro X-ray Fluorescence (μ-XRF) and Handheld-XRF Characterization (hh-XRF)

The CuO/PMMA-coated Si substrates were also characterized via μ-XRF in order to determine whether the CuO nanofillers were homogeneously dispersed within the PMMA matrix or whether they only form agglomerates, as seen through optical and electron microscopy. Such an approach, to the best of our knowledge, has not been attempted before.

The μ-XRF spectrometer probe used in this work consists of a micro focus Rh-anode tube, a polycapillary X-ray lens as a focusing optical element (IfG-Institute for Scientific Instruments GmbH, Berlin, Germany), with a focal distance equal to 21.2 mm, and a nominal gain factor that varies between 3625–4900–1200 for energies within the intervals of 3–5, 10–15 and 25–30 keV, respectively. The X-ray detection channel consists of an electro-thermally cooled 10 mm^2^ silicon drift detector (X-Flash,1000 B) with full width at half maximum at 5.89 keV equal to 146 eV at 10 kcps coupled with a digital signal processor. Three different stepping motors, coupled with the spectrometer head, allows for its three-dimensional movement, facilitating the elemental mapping studies. Finally, a color charge-coupled device camera (x13), a dimmable white light-emitting diode for sample illumination and a laser spot assist in the documentation and sample alignment. The spectrometer spatial resolution (FWHM) for the excitation of the Cu-Kα line was measured to be ~80 µm. For Cu quantification purposes, the μ-XRF spectrometer was calibrated by means of a multi-elemental, nm-scaled sample manufactured by AXO-Dresden, GmbH. The stratified sample was composed of ~10 nm individual layer thicknesses of Cr, Al, Ni, Cu and Ti elements deposited on a few micrometers polymer film. This reference sample belongs to the same batch of similar reference materials developed for synchrotron radiation experiments [36]. The X-ray tube measurement conditions were set at 50 kV, 600 µA using an unfiltered exciting beam, whereas the μ-XRF scanning parameters were set as follows: step size 0.1 mm, 20–25 s measurement time per step with a typical investigated sample area of about 1.5 × 1.5 mm^2^. For certain samples, a larger area was scanned. The spectrum deconvolution and quantification were carried out using the PyMca analysis software [37]. Si substrates coated with the 4% *w*/*v* PMMA solutions at all 3 rotation speeds were used as references.

In addition to the scanning μ-XRF measurements, a hh-XRF analyzer with Rh anode transmission X-ray tube (Tracer 5i, Bruker) was used to perform selected screening measurements of the average Cu deposited areal density. The hh-XRF measurements were performed at 30 kV/110 μA high voltage/current operating conditions using the combined Ti/Al filter for the exciting X-ray beam provided by the manufacturer.

## 3. Results and Discussion

### 3.1. Phase 1

**Figure 2:**
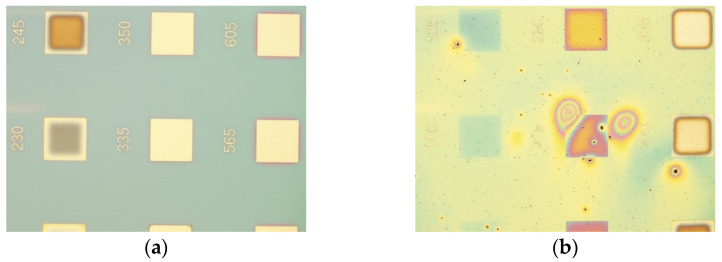

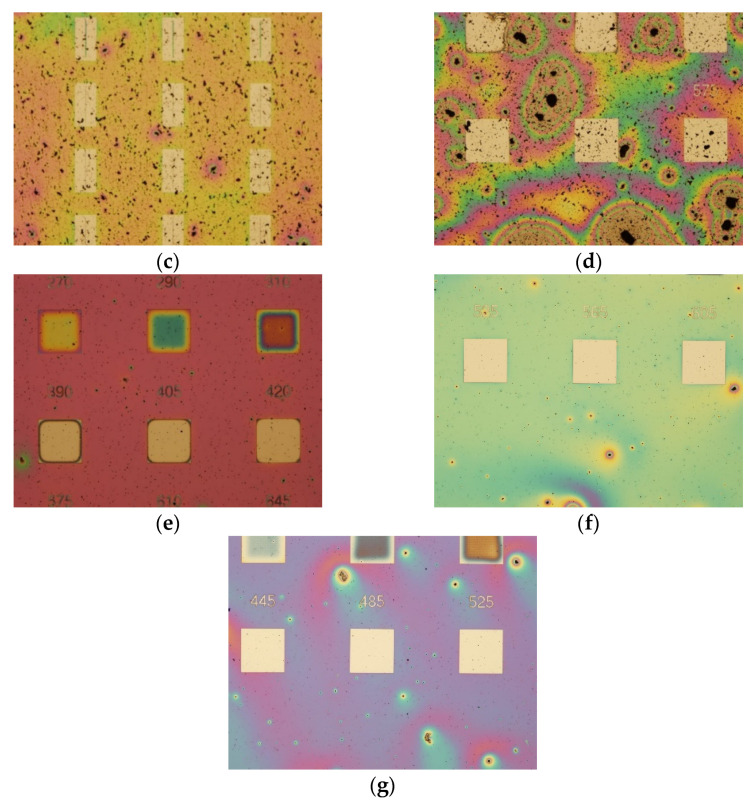
Optical microscope images (magnification: ×10) from the contrast curve patterns used to determine the suitability of the CuO/PMMA nanocomposites as resist materials. The square patterns have a size of 200 μm × 200 μm (detailed description can be found in Section 2 of SM). Analytically, (**a**) REF-1k, (**b**) 1%CuO-1k, (**c**) 2%CuO-1k (picture of resolution structures to demonstrate their characteristic pattern), (**d**) 3%CuO-1k, (**e**) 1%CuO-1k-DF, (**f**) 1%CuO-3k, and (**g**) 1%CuO-4k.

As a first step, the EBL patterned samples were examined under an optical microscope (Figure 2). It was readily observed that increasing the concentration of CuO to 2% or 3% *w*/*v* resulted in the formation of large agglomerates that reached up to 100 μm (Figure 2c,d) in size, rendering these concentrations unsuitable for the production of CuO/PMMA nanocomposites. The agglomerates were so large that they prevented obtaining accurate and reliable stylus profilometry measurements necessary for the determination of resist thickness. In addition, it was also observed that the agglomerates were not successfully removed from the exposed areas after the EBL development step. For those reasons, samples 2%CuO-1k and 3%CuO-1k were not included in the subsequent analysis of the films with respect to their performance as resists and were deemed unsuitable for any practical application of the nanocomposites. However, it is worth noting that it was still possible to pattern the nanocomposite films with e-beam lithography regardless of the agglomerates suggesting that it might still be possible to use solutions of higher nanofiller loadings after filtration. This aspect has not been addressed in this work and is still under investigation.

**Figure 3:**
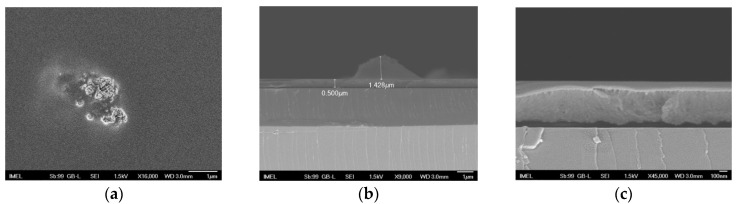
SEM images of sample 1%CuO-1k (**a**) top-down view showing a typical agglomeration of nanofillers found in the film, (**b**) cross-section of one of the nanofiller agglomerates demonstrating their relative size compared to the nanocomposite film thickness and (**c**) cross-section and magnification of the nanocomposite film at a section devoid of agglomerates. Scale bars: 1 μm in (**a**,**b**); 100 nm in (**c**).

In contrast, when the concentration of the nanofillers was kept to 1% of the agglomerates were significantly smaller in size (Figure 2b,c,f,g), but still their size, as shown after SEM inspection, could reach up to 1–3 μm (Figure 3a,b). The scattered agglomerates would protrude through the film (Figure 3b), demonstrating the need to find a way to hinder their formation. The rest of the film exhibited great homogeneity (Figure 3c), but it was not possible to determine, through SEM, whether individual nanofillers were dispersed within the film.

During the experiments, it was observed that, after approximately 24 h, the nanofillers would sediment in all CuO/PMMA solutions (Figure S5b). The inability to form stable inorganic/polymer solutions is one of the main challenges of polymer nanocomposites. Therefore, in order to address this issue and to find a solution, which would still keep the suggested methodology as simple and as cost-efficiently as possible, a deflocculant was used to examine whether it would extend to the stability of the solutions. The chosen deflocculant was Darvan C, a substance commonly employed in ceramic dispersions giving low viscosity slip and low foam production [38,39,40]. Upon the addition of Darvan C, the solution turned into a stable emulsion and no signs of sedimentation were observed for several days. Over the course of a week, light blue sediments appeared on the vial walls, an indication that copper (II) hydroxide salts were formed due to the presence of the amine groups of Darvan C (Figure S5c). After a month, most of the copper oxide had turned into copper (II) hydroxide dehydrate, as attested by the light-blue co-aggulated sediment and the clear color of the solution, similar to that of pure PMMA (Figure S5d). This suggests that, even though immediate sedimentation was prevented, the CuO/PMMA/DF nanocomposites have a shelf-life of approximately 1 week, which is still an improvement to the limited shelf-life of 24 h in the absence of deflocculant. As far as the film is concerned, it still contained agglomerates that were visible through the optical microscope (Figure 2e), which, however, were more uniform in size and more homogeneously dispersed compared to all the other films.

Resist variations were evaluated in terms of their bulk lithographic properties (thickness, sensitivity, and resolution) via contrast curve data analysis and optical inspection. Spin curves (resist thickness after development versus spin speed) were constructed from profilometric measurements of the contrast curve patterns.

**Figure 4:**
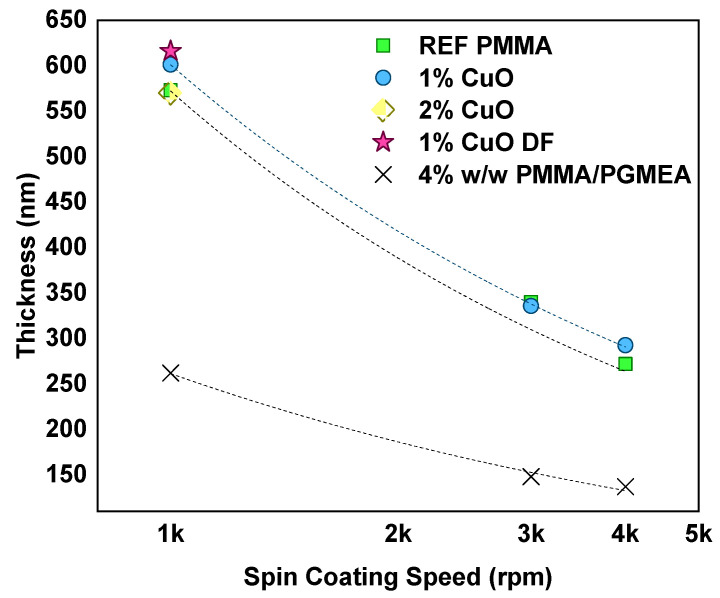
Spin curves for the “bare” PMMA reference samples (green squares), the nanocomposites containing 1% *w*/*v* CuO (blue circles), sample 2%CuO-1k (yellow diamond) and 1%CuO-1k-DF (magenta star). Crosses correspond to the 4% *w*/*w* PMMA/PGMEA.

As a first observation, it was seen that the films produced, both the reference and nanocomposite ones, were considerably thicker compared to films produced under the same conditions by 4% *w*/*w* PMMA in PGMEA, as described in Section 2.3 (even though the authors acknowledge that 4% *w*/*w* PMMA in PGMEA is not exactly the same as a 4% *w*/*v* PMMA/PGMEA/Acetone solution, it is the closest in terms of a reference resist). 4% *w*/*w* PMMA in PGMEA solutions typically provide films with a thickness of 260 nm, 150 nm and 130 nm after PAB, when resist-spinning is performed at 1k, 3k and 4k rpm, respectively (Figure 4). It is postulated that acetone, as a more volatile solvent with respect to PGMEA, evaporates much faster during the spin-coating process, resulting in thicker films. Moreover, the addition of a different solvent has a direct effect on the viscosity, which, in this case, seems to be increased. Turning the focus on the nanocomposite films, the addition of nanofillers with 1% *w*/*v* loading slightly increased the thickness of produced films (Figure 4). The presence of deflocculant even further increased the thickness of the nanocomposite film suggesting, in a very indirect way, that its presence might have assisted in the nanofiller distribution within the polymer matrix.

**Figure 5:**
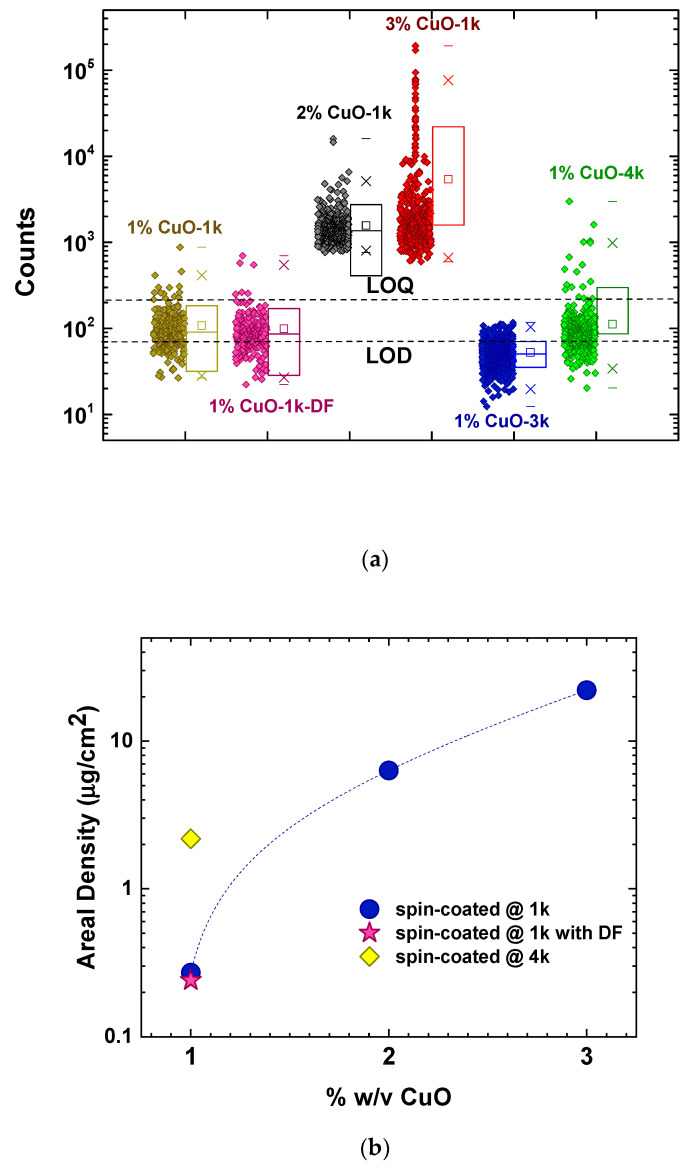
Synopsis of μXRF measurements for all CuO/PMMA samples (Phase 1). (**a**) Scattered solid diamonds depict the intensity signal from each point (pixel), while open squares show the average signal value. The large rectangles show the range between 25% and 75% of the maximum obtained signal, the crosses mark the range between 1% and 99% of the maximum obtained signal and the horizontal dashes show the minimum and maximum obtained values. Dashed lines show the limit of detection (LOD) and the limit of quantification (LOQ) set as 3*LOD. (**b**) Cu areal density as a function of the nanofiller loading and the preparation parameters. Blue circles: films of various loadings spin-coated at 1 krpm; Magenta star: sample 1%CuO-1k-DF with 1% CuO nanofillers and deflocculant spin-coated at 1 krpm; Yellow diamond: sample 1%CuO-4k with 1% CuO nanofillers and spin-coated at 4 krpm.

In an effort to assess whether the films contained dispersed nanofillers and not only agglomerates, and to substantiate the validity of the assumptions above, the samples were investigated by scanning μ-XRF measurements. The pixel Cu-Kα μ-XRF data are summarized in Figure 5 and have all been deduced by means of the PyMca software accounting for rather negligible blank contributions. As readily seen, the films with 2% and 3% *w*/*v* CuO loadings yielded signals that were two orders of magnitude larger than all samples of 1% *w*/*v* loading. The signals also varied by two orders of magnitude from point to point across the samples, a fact that it is in accordance with the presence of the large agglomerates, as well as the smaller agglomerates that spot the surface observed through optical microscopy (Figure 2c,d). All the samples with 1% *w*/*v* nanofiller loading exhibited more or less the same behavior. A sizeable percentage (10−25%) of the signals were below the limit of detection (LOD), the majority of the signals (85−70%, respectively) were between the LOD and the limit of quantification (LOQ) was set as 3*LOD, and only 5% were above LOQ, most likely related to the presence of large agglomerates. However, the signals also spanned across two orders of magnitude, suggesting that individual nanofillers were most likely dispersed within the volume of the polymer matrix, but their small size and spatial distribution results into low signals. It should be noted that sample 1%CuO-3k yielded results below or very close to the LOD and was not further assessed with this particular method.

Despite the fact that most of the signals were below the LOQ, an attempt was made to calculate the areal density (Figure 5b; Table 3). It should be clarified that the Cu areal densities are reported for the whole scanned area by summing all the individual pixel (0.1 mm) size measurements. The LoD for Cu areal density, as obtained from the sum spectrum, is in the order of 0.02 µg/cm^2^ corresponding to a measurement time of about 5000 s. The theoretical calculation for the expected Cu areal density of the films taking into account their thickness and nanofiller loadings would be in the order of 0.2–0.9 µg/cm^2^ (values given in parentheses in Table 3). For the 1% *w*/*v* nanofiller loadings, the experimental values were within the same order of magnitude with the theoretical expectations, but for the 2% *w*/*v* loading, the measured areal density was increased by two orders of magnitude, corroborating with the scenario of aggregate formation and their inhomogeneous dispersion within the polymer.

**Figure 6:**
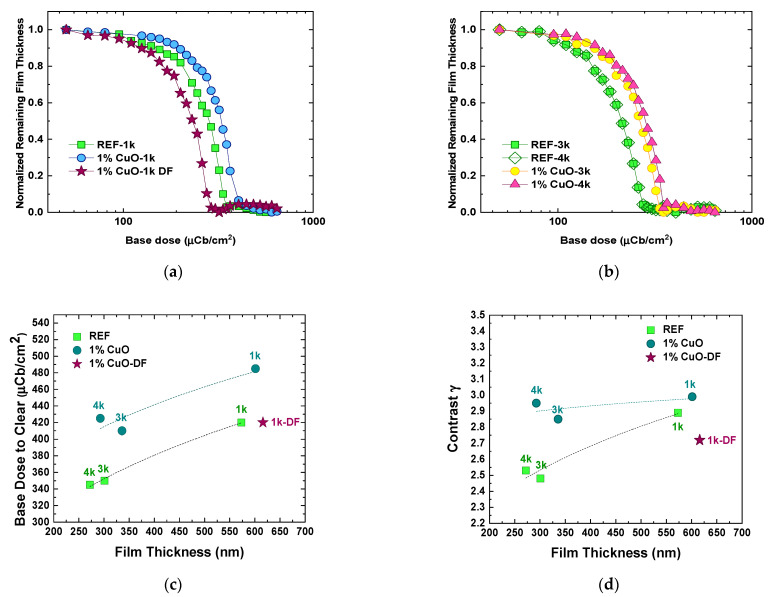

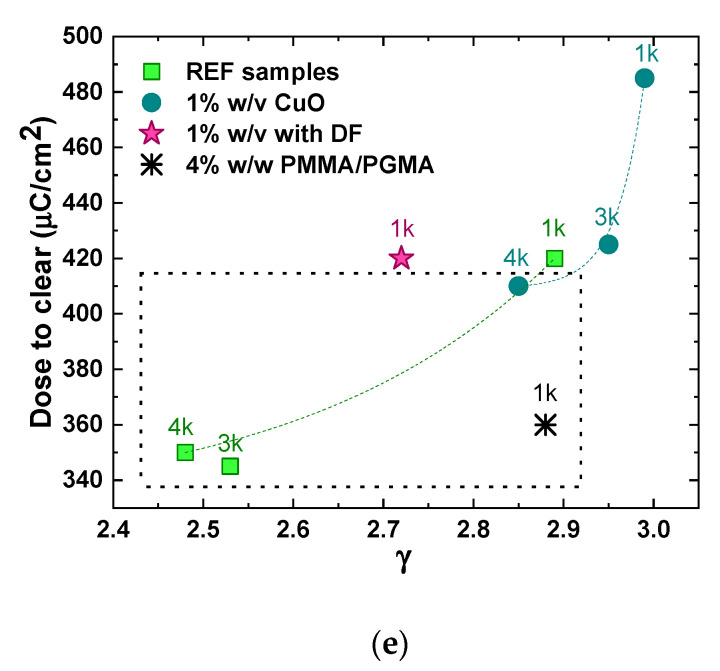
Contrast curves (Normalized Remaining Thickness as a function of the base dose) for patterned samples with films spin-coated at (**a**) 1000 rpm, (**b**) 3000 and 4000 rpm; (**c**) sensitivity (expressed as the minimum DTC) versus film thickness, (**d**) contrast γ versus film thickness, and (**e**) dose-to-clear versus contrast γ for all nanocomposite films of Phase 1 that were evaluated as EBL resists. The dotted rectangle includes resist films of similar thicknesses of ~260–300 nm. Dashed lines in graphs of (**c**–**e**) serve only as a guide to the eye, while labels indicate the spin-coating rotation speed in rpm.

As a final step of this phase, the contrast patterns of the films were evaluated. The behavior of the nanocomposite films is illustrated in Figure 6a,b. Sensitivity (expressed as a dose to fully clear the resist film, including remains at the corners of the exposed square) and contrast γ are demonstrated in Figure 6c,d, as a function of the films’ thickness and preparation conditions. The results are summarized as dose-to-clear (DTC) versus contrast γ in Figure 6e, since the reference and their respective nanocomposite films had comparable thicknesses (Figure 4). For the reference samples, DTC was between 345 µC/cm^2^ and 420 µC/cm^2^, while upon addition of the nanofillers, DTC appreciably increased, ranging from 425 µC/cm^2^ to 485 µC/cm^2^, indicating that the presence of the CuO nanofillers affect the sensitivity of the resist matrix. Contrast γ values are within the range of 2.5–2.9 for all the samples. For the “standard” 4% *w*/*w* PMMA/PGMEA, the associated DTC for resist films of ~260 nm (spin-coated at 1k rpm) is in an average 360 µC/cm^2^ and contrast γ is ~2.9 (Figure 6e), when EBL is performed at 100 kV and the development duration is set to 60 s.

It appears that the addition of acetone to PMMA/PGMEA (REF samples) slightly decreases contrast γ without affecting DTC. According to Gaikwad et al. [41], acetone is a relatively good solvent for PMMA, and in this work, it did not drastically affect the properties of PMMA as a positive tone resist in terms of contrast γ and DTC. Upon addition of nanofillers, DTC increased significantly, while contrast γ returned to the value of 2.9–3.0. The increase of DTC is attributed to scattering of the beam electrons by the nanofillers and serves as an indirect verification that the metal oxide nanoparticles, even though not directly observable by means of microscopy, are indeed dispersed within the polymer matrix. In contrast to the results in References [16,17], according to which the presence of 1 nm silica nanoparticles in ZEP520^®^ increased the resolution of the resist without sacrificing the sensitivity and contrast, the presence of the CuO nanofillers in PMMA in this work decreases the sensitivity, but slightly increased the contrast with respect to the reference samples (Figure 6e). This can be attributed to the larger size of the CuO nanofillers (~10 nm), which can scatter the electrons more efficiently than the 1 nm-wide silica nanoparticles. It is also suggested that the presence of agglomerates might also play an additional role in electron scattering. The slight increase of γ with respect to the reference samples might also be due to the role of the nanofillers as scattering centers, which do not just reduce the penetration depth of the incident electrons (increasing thus DTC), but also their lateral range and the spreading of secondary electrons [16].

Notably, the addition of the deflocculant “returned” the behavior of the nanocomposite closer to that of bare PMMA in terms of DTC. This behavior is suggestive that the deflocculant, apart from the prolonged shelf-life of the CuO/PMMA solutions, may offer a better homogenization of the nanocomposite and a more uniform dispersion of the nanofillers within the PMMA matrix. If the nanofillers are more uniformly dispersed (as already suggested by optical microscopy) scattering of the electrons may not be as profound as in the case of sample 1%CuO-1k, which is “dotted” with larger agglomerates and the film as a resist requires approximately the same base dose to be fully cleared as the bare PMMA.

The findings of Phase 1 are summarized in Table 3.

Phase 1 proved that the present methodology, despite its simplicity, can be used to produce CuO/PMMA nanocomposites as positive tone EBL resist materials. The results suggested that nanofiller loadings should not exceed 1% *w*/*v*, while it is important to more efficiently control the formation of metal oxide aggregates. The use of the deflocculant significantly extended the shelf-life of the CuO/PMMA and offered an improved homogenization and nanofiller dispersion within the polymer matrix. These results led to Phase 2, which concentrated on examining four parameters:

(1) In order to improve the nanofiller dispersion, the volume of the additional solvent was increased. For that reason, the initial PMMA/PGMEA concentration was slightly increased, from 5% *w*/*w* to 6% *w*/*w*, to allow for a larger amount of solvent to be added to reach the final 4% *w*/*v* PMMA concentration in the CuO/PMMA solution.
(2) One more solvent, MEK, was tested in conjunction to acetone to test the role of the solvent
(3) The effect of the deflocculant was re-examined with the new conditions of increased solvent volume and the new solvent
(4) The effect of filtration prior to spin-coating deposition of the resist films in the absence of DF was tested.

### 3.2. Phase 2

As already described in Section 2 and summarized in Table 2, eight resist films were prepared and patterned by EBL, four for each one of the two solvents. The CuO loading was set to 1% *w*/*v* and the PMMA concentration to 4% *w*/*v*, both with respect to the final solution volume. The spin-coating rotation speed was fixed to 1000 rpm. XRF measurements, EBL patterning and subsequent contrast curve and resolution analysis was conducted in exactly the same manner as in Phase 1. The contrast patterns of the samples are shown in Figures S6 and S7, while the resolution patterns are shown in Figures S8 and S9. All data compiled from Phase 2 are summarized in Table 4.

Contrary to what was expected, the increased amount of solvent did not ameliorate the nanofiller dispersion, but instead resulted in the formation of more and larger aggregates. Both samples, ACE-CuO and MEK-CuO, could not be fully assessed in terms of their sensitivity due to the large number of aggregates that did not allow us to obtain reliable stylus profilometry measurements for all base doses and construct a contrast curve that could be analyzed. The data collected have only been used to calculate the film thickness and standard deviation after the EBL, but not to calculate the sensitivity of the film. Nonetheless, the resolution patterns were inspected to form a more complete picture of their behavior. The reason behind the formation of numerous aggregates with the increase of the solvent volume is still under investigation, but an initial assumption is that the lower viscosity does not enhance the miscibility, but instead promotes phase separation of the constituent materials.

**Figure 7:**
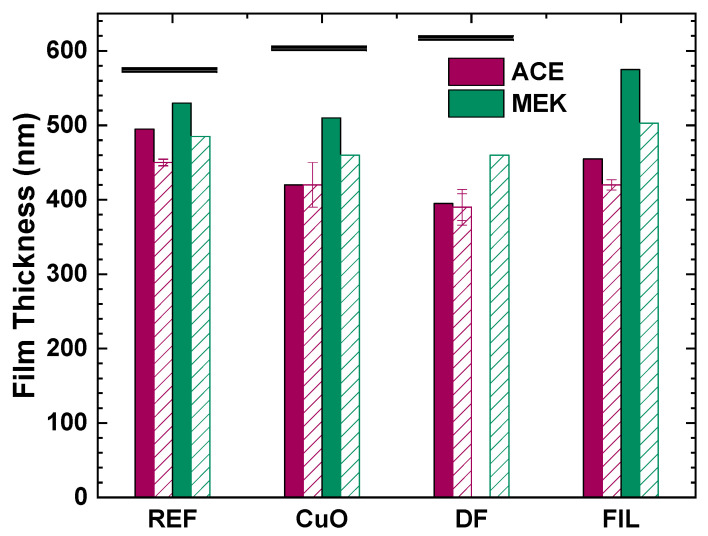
Film thickness of Phase 2 CuO/PMMA nanocomposite films, as measured by ellipsometry prior to EBL (solid bars) and as calculated by stylus profilometry of the contrast patterns after development (striped bars). Magenta bars correspond to acetone-based nanocomposites; green bars correspond to MEK-based nanocomposites. Horizontal black lines denote the film thickness of the respective samples from Phase 1.

The film thicknesses, as measured by ellipsometry, with respect to the preparation conditions (before EBL exposure), are presented in Figure 7 alongside the average film thickness and its standard deviation, as measured from the stylus profilometry after development of the film. All films were thinner with respect to the Phase 1 samples, which means that the viscosity was reduced. The acetone-based films were, in all cases, slightly thicker than the MEK-based ones. When MEK was employed, the addition of the deflocculant resulted in the thinnest films. The filtration resulted in films comparable in thickness to the reference ones, regardless of the solvent.

**Figure 8:**
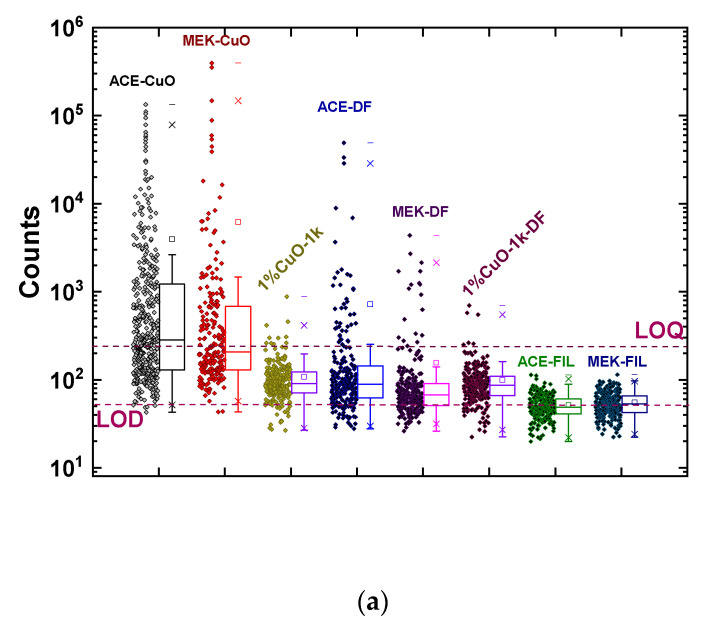

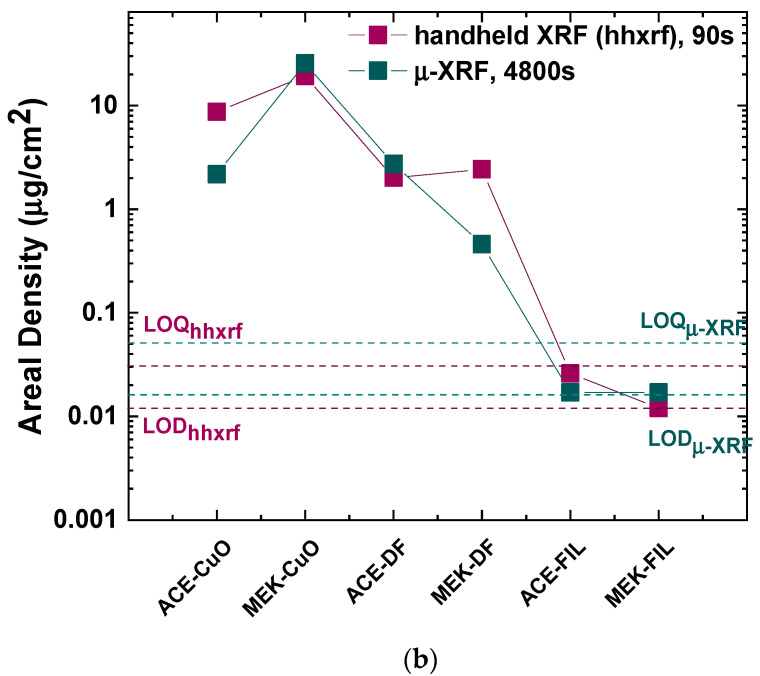
(**a**) Synopsis of μ-XRF measurements for all Phase 2 CuO/PMMA samples. The corresponding measurements of 1%CuO-1k and 1%CuO-1k-DF from Phase 1 have been included for comparison. Scattered solid diamonds depict the intensity signal from each point, while open squares show the average signal value. The large rectangles show the range between 25% and 75% of the maximum obtained signal, the crosses mark the range between 1% and 99% of the maximum obtained signal and the horizontal dashes the minimum and maximum obtained values. Dashed lines show the limit of detection (LOD) and the limit of quantification (LOQ) set as 3*LOD. (**b**) Comparison of the calculated Cu areal density by the hh-XRF analyzer (magenta bars) and the μ-XRF spectrometer system (green bars). Dashed lines denote the LOD and LOQ determined for measurement times of 4800 s and 90 s for the μ-XRF and hh-XRF spectrometers, respectively.

The μ-XRF measurements corroborated the fact that increasing the amount of solvent resulted in an increased number of aggregates, as well as aggregates of larger size (Figure 8a). When the results of ACE-CuO and MEK-CuO are compared to the corresponding results of 1%CuO-1k, it is readily seen that there was a significant increase in the signal intensity dispersion over three orders of magnitude with more than 50% of the signals being over the LOQ affirming the formation of larger aggregates. When a deflocculant was added the signal dispersion was decreased and most of the signals were in the same order of magnitude, again corroborating the optical microscopy and profilometry observation—as well as the results of Phase 1—that the presence of the deflocculant limits the formation of aggregates and improves the miscibility of the nanofillers without completely eliminating the aggregate presence. When comparing ACE-DF and MEK-DF to 1%CuO-1k-DF, it was again observed that the increase in the solvent content resulted in an increase in aggregate number and size. When filtration was applied, the μ-XRF signals dropped close to or even below the LOD of the method and it was not possible to affirm the presence of nanofillers within the resist film or to safely deduce any conclusion. For that reason, additional measurements were performed with the hh-XRF analyzer, offering the possibility to deduce average Cu areal densities over a large irradiated area (approximately described as a circle with 8 mm in diameter, Figure 8b). In fact, the hh-XRF analysis of the ACE-FIL sample succeeded to detect a minimum, but not quantifiable amount of Cu above the respective LOD. However, the non-consistent Cu areal densities deduced by the μ-XRF and hh-XRF spectrometers (ACE-CuO and MEK-DF in Figure 8b) might be due to the inhomogeneity of the deposited area, as the μ-XRF analyzed ~2.25 mm^2^ versus the ~47.1 mm^2^ probed by the hh-XRF analyzer. Comparing the theoretical values to the experimental values, one readily observes that the Cu areal density exceeds by one or two orders of magnitude the anticipated areal density that would correspond to the nominal nanofiller loading of 1% *w*/*v* (~0.3–0.4 µgr/cm^2^), implying that the nanofillers have aggregated into larger structures non-homogeneously dispersed within the solution. Applying filtration resulted in areal densities very close to the LOD and one order of magnitude lower than the one corresponding to 1% *w*/*v*, suggesting that the aggregates were retained in the filter and only a smaller quantity than 1% *w*/*v* of nanofillers were present in the resist film.

**Figure 9:**
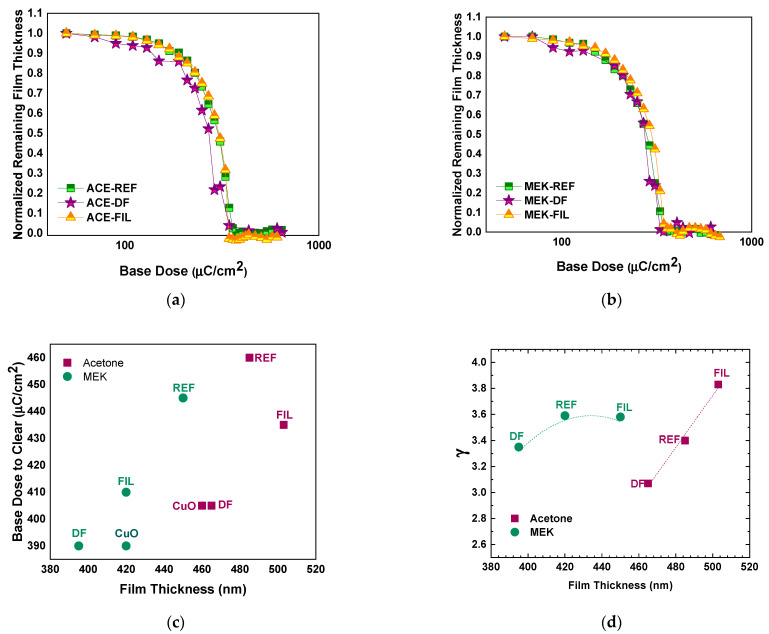
(**a**,**b**) Contrast curves (Normalized Remaining Thickness as a function of the base dose) for the acetone-based and MEK-based nanocomposite films, respectively. (**c**) Sensitivity expressed as minimum base dose-to-clear versus film thickness and (**d**) contrast γ versus film thickness for all nanocomposite films of Phase 2. Lines serve only as a guide to the eye.

The contrast curves and subsequent sensitivity and contrast γ analysis was performed based on the thickness measured after development. The analysis (Figure 9) demonstrated that, when the CuO nanofillers are added to the PMMA, the DTC drops with respect to the bare PMMA, as was also observed in Phase 1. This further adds to the scenario that the electrons scatter on the nanofillers. This is further substantiated by the fact that the lowest DTCs are observed for the nanocomposites containing the deflocculant; despite the improved homogenization and stability, the deflocculant cannot prevent the formation of agglomerates, which act as large scattering centers. On the contrary, when the CuO/PMMA solution is filtered most of the agglomerates do not end up in the film, and the dose-to-clear increases with respect to the nanocomposites with deflocculant. Still, the presence of nanofillers and the related electron scattering maintain DTC levels to lower values with respect to the reference films (Figure 6c–e). When comparing the two solvents, the dose-to-clear is in general lower for the case of MEK, a fact that is expected, given that the resulting resist films are thinner to begin with.

As far as contrast γ is concerned, in accordance with the results of Phase 1, the addition of the deflocculant decreases its value compared to all other samples, irrespective of the solvent used. Nonetheless, for the case of MEK, all resists had γ values that did not differ considerably among them (Figure 6d,e). In contrast, the γ values for the acetone-based films had considerable variations among them. Additionally, γ for the acetone-based resists was increased compared to Phase 1 samples. Notably, γ between ACE-DF and ACE-FIL differed by 0.7 (an increase of almost 25%).

The resolution patterns of acetone and MEK based CuO/PMMA nanocomposite films (Figures S8 and S9) show that the addition of the nanoparticles have an observable influence on the resolution of the protruding ridges, while the resolution of grooves does not change (300 nm for all samples). We believe that this phenomenon is caused by the loss of the adhesion between the film and the substrate due to the nanofillers size. In the case of films with only the nanofillers and without filtration, no protruding ridges, even for 20 µm lines, could be formed, although it is clear that, when the film is underexposed, the line is there, but when DTC is reached, the line loses its adhesion to the substrate. This fact is minimized by the addition of deflocculant, which offered better homogeneity and smaller nanofillers size, resulting in better adhesion to the substrate. The same stands for the filtration of the solution; all the large size nanofillers are retained in the filter, therefore smaller nanofillers are in the film, resulting again in a better adhesion to the substrate.

A very significant difference between the two solvents was that the MEK-based films were extremely sensitive to the base dose and could only be reliably patterned when the exposure dose was close to DTC (Figure S9). For doses slightly higher than DTC, it was not possible to create protruding ridges smaller than 20 µm, while the grooves, when the line width was smaller than 1 µm, appeared to not be fully exposed. For that reason, MEK was proven to be an unsuitable solvent for this particular methodology. However, acetone resulted in films with increased tolerance to exposure doses (Figure S8), although the resolution limits (300 nm for grooves and 5 µm for protruding ridges) are still rather large for the actual capabilities of EBL. It appears—and in the most dramatic way, this was observed through the reference samples that do not contain any nanofillers—that the addition of the solvent (either MEK or acetone) affects the lithographic properties of PMMA in a profound way, mostly in terms of resolution. Therefore, it is of paramount importance to optimize the quantity of the solvent that may be added to the initial PMMA/PGMEA solution to incorporate the CuO nanofillers.

Summarizing the findings of this study, the first general observation is that, despite its simplicity and “crudeness”, the suggested methodology can result in principle in CuO/PMMA nanocomposites that can be patterned with EBL and thus be employed in the future for polymer nanocomposite-based nanodevices. The results showed that nanofiller loadings should not exceed 1% *w*/*v* due to the formation of large metal oxide aggregates. EBL of the CuO/PMMA films showed that the presence of the CuO nanofillers, as well as the addition of solvent (either acetone or MEK), both affect the properties of PMMA by increasing the DTC and its contrast, but putting a significant limit on the resolution. In particular, the results of Phase 2, where the initial PMMA/PGMEA solution concentration was increased from 5% to 6% *w*/*w* and the amount of solvent was increased, it was observed that there was a deterioration in the resist resolution (Figures S9 and S10) and a more pronounced aggregation of the nanofillers (Figure 8a), as indicated by the μ-XRF measurements and the calculated CuO areal density (Table 1 vs. Table 2). This indicates that the role of the solvent used to mix the nanofillers into the PMMA/PGMEA and its volume ratio with respect to the initial PMMA/PGMA solution is critical in controlling the properties of the nanocomposite as a resist. Future studies will focus on determining the optimum volume ratio of the added solvent to PMMA/PGMA that will not have a deleterious effect on the resolution of the resist, while minimizing the nanofiller aggregation.

In addition, Phase 2 revealed that MEK is not a suitable choice for this method. Despite the fact that the CuO/PMMA nanocomposites realized using MEK could be patterned by EBL, scrutinizing the resolution patterns revealed that the films were very sensitive to the exposure dose and patterns could only be reliably produced when working very close to DTC, which is a non-desirable feature for any resist. Furthermore, μ-XRF revealed that using MEK leads to the formation of very large aggregates, as indicated by the increase in the CuO areal density by three orders of magnitude (25.5 µg/cm^2^ instead of the anticipated 0.34 µg/cm^2^).

Further, this study indicated that the use of a deflocculant offers an improved homogenization and nanofiller dispersion within the polymer matrix and significantly extends the shelf-life of the CuO/PMMA, from 1 day to 1 week. Therefore, it is imperative that the deflocculant is used. Again, additional studies are required to optimize the amount of deflocculant that would further improve the homogeneity of the nanocomposite, the nanofiller dispersion within the polymer host and possible extension of the shelf-life of the solutions. Filtration ameliorated the films by removing the large aggregates, but unless the initial solutions are more homogenized and with improved nanofiller dispersion, filtration by itself is not enough. Hence, further works studying the combined effect of deflocculant use and filtration are required.

## 4. Conclusions

In this work, the demonstration of an easy and low-cost method to produce e-beam resist materials based on CuO/PMMA nanocomposites was accomplished, showing all the steps from the synthesis of the CuO nanofillers to the development and the evaluation of lithographic performance of the resist. It was established that the suggested method, despite its simplicity, can produce CuO/PMMA nanocomposite EBL resists that can be used in the future for several applications and nanodevices. This study revealed that the most critical parameters are the nature and the volume ratio of solvent with respect to the original PMMA solution, which control both the resolution of the nanocomposite resist and the nanofiller homogeneous dispersion. It was also established that the use of a deflocculant is necessary in order to improve the dispersion of the nanofillers, the homogeneity of the nanocomposite solutions and to extend their shelf-life. Filtration of the resist solutions prior to EBL may be beneficial so as to remove the remaining nanofiller aggregates. Finally, apart from the proof-of-concept of the suggested methodology, this work demonstrated that μ-XRF—which was used for the first time according the authors’ knowledge in such a context—is a powerful alternative method for the non-destructive and time-efficient characterization of polymer nanocomposite films offering meaningful insights and quantifiable observations. Future works will concentrate on optimizing the key parameters of the methodology, testing of other solvents apart from acetone and MEK, and most importantly extending it to other types of metal oxide nanofillers.

## Figures and Tables

**Table 1:** Phase 1 samples coated with CuO/PMMA polymer nanocomposite solutions. REF-Yk correspond to films prepared by the 4% *w*/*v* PMMA-acetone solution without any nanofillers spin-coated at Y krpm. X%CuO-Yk correspond to films prepared containing X% *w*/*v* CuO nanofillers spin-coated at Y krpm. DF denotes the addition of deflocculant.

Sample Name	CuO Concentration (*w*/*v*)	Deflocculant	Spin Coating Speed (rpm)	Thickness (nm)
REF-1k	0%	NO	1000	573
REF-3k	0%	NO	3000	340
REF-4k	0%	NO	4000	272
1%CuO-1k	1%	NO	1000	601
1%CuO-3k	1%	NO	3000	336
1%CuO-4k	1%	NO	4000	293
2%CuO-1k	2%	NO	1000	570
3%CuO-1k	3%	NO	1000	N/A
1%CuO-1k-DF	1%	YES	1000	616

**Table 2:** Phase 2 samples coated with CuO/PMMA polymer nanocomposite solutions. Prefix ACE corresponds to solutions prepared with acetone as the solvent, while prefix MEK corresponds to solutions prepared with MEK as the solvent. DF denotes the addition of deflocculant and FIL denotes that the solution was filtered.

Sample Name	CuO Loading	Solvent	Deflocculant	Filtering
ACE-REF	0%	Acetone	NO	NO
ACE-CuO	1%	Acetone	NO	NO
ACE-DF	1%	Acetone	YES	NO
ACE-FIL	1%	Acetone	NO	YES
MEK-REF	0%	MEK	NO	NO
MEK-CuO	1%	MEK	NO	NO
MEK-DF	1%	MEK	YES	NO
MEK-FIL	1%	MEK	NO	YES

**Table 3:** Summary of Phase 1 Results.

Sample Name	Film Thickness (nm)	Dose-to-Clear (μC/cm^2^)	γ	XRF Areal Density (μgr/cm^2^)
REF-1k	575	420	2.89	N/A ^a^
REF-3k	340	350	2.48	N/A
REF-4k	270	345	2.53	N/A
1%CuO-1k	600	485	2.99	0.27 (0.48) ^b^
1%CuO-3k	335	410	2.85	−(0.26)
1%CuO-4k	290	425	2.95	0.30 (0.23)
1%CuO-1k-DF	615	420	2.72	0.24 (0.49)
2%CuO-1k	570	N/A	N/A	6.3 (0.91)
3%CuO-4k	N/A	N/A	N/A	22.1 (-)

^a^ N/A: non-applicable. ^b^ Values in parentheses are the theoretical calculations of the Cu areal density.

**Table 4:** Summary of Phase 2 results.

Sample Name	Film Thickness ^a^ (nm)	Dose-to-Clear (μC/cm^2^)	γ	Resolution ^b^ (groove/ridge)	XRF Areal Density (μgr/cm^2^) ^e^
ACE-REF	530/485	460	3.4	300 nm/300 nm	N/A
ACE-CuO	510/460	405	N/A	300 nm/10 μm	2.2/8.7 (0.37)
ACE-DF	-/465	405	3.1	300 nm/5 μm ^c^	2.7/2.0 (0.37)
ACE-FIL	575/503	435	3.8	300 nm/10 μm ^c^	-/0.02 (0.40)
MEK-REF	495/450	445	3.6	300 nm/300 nm	N/A
MEK-CuO	420/420	390	N/A	300 nm/-	25.6/19.3 (0.34)
MEK-DF	395/395	390	3.3	300 nm/5 μm ^d^	0.46/2.4 (0.32)
MEK-FIL	455/420	410	3.6	300 nm/5 μm ^d^	-/0.04 (0.34)

^a^ First value corresponds to ellipsometry result after PAB; second value to stylus profilometry of contrast patterns after development. ^b^ The first number indicates the minimum feature size achieved for the formation of grooves, while the second for protruding ridges. ^c^ Recorded resolution achievable only for moderate doses close to the minimum dose-to-clear–High sensitivity to overdose. ^d^ Recorded resolution achievable only for dose to clear—higher doses result in no ridges. ^e^ First value obtained by μ-XRF/ Second value obtained by the hh-XRF analyzer; Values in parentheses are the theoretical calculations of the Cu areal density.

## Supplementary Materials

The following are available online at https://www.mdpi.com/2079-4991/11/3/762/s1, Figure S1: Schematic representation of the CuO Nanofiller Synthesis, Figure S2: SEM images of the nanopowders produced with copper (II) acetate monohydrate concentration of (**a**) 30 mM, (**b**) 65 mM and (**c**) 100 mM. Subscripts denote the temperature of synthesis: (1) 70 °C, (2) 80 °C and (3) 90 °C. Scale bar in all images: 100 nm; magnification: ×100,000, Figure S3: (**a**) XRD spectra of the nanopowders compared to the monoclinic phase of CuO (JCPDS pattern no 45-09370) demonstrating the pure monoclinic phase of all samples; (**b**) Crystallite size as function of copper (II) acetate monohydrate concentration for the three synthesis temperatures of 70 °C (black squares), 80 °C (red circles) and 90 °C (blue triangles), Figure S4: Schematics of (**a**) the contrast curve patterns (top view), (**b**) the resolution patterns (top view), and (**c**) the cross-section of the resolution patterns showing the wells and ridges, Figure S5: Photographs of the CuO/PMMA solutions (**a**) 1%CuO/PMMA after production and just prior to use for sample spin-coating, (**b**) 1%CuO/PMMA after 48 h stored in ambient conditions, (**c**) 1%CuO/PMMA with deflocculant after 2 weeks stored in ambient conditions, and (**d**) 1%CuO/PMMA with deflocculant after 1 month stored in ambient conditions, Figure S6: Optical microscope images (×10) of the contrast patterns for the acetone-based CuO/PMMA nanocomposite film, Figure S7: Optical microscope images (×10) of the contrast patterns for the MEK-based CuO/PMMA nanocomposite films. Base doses are indicated on the left-hand side, Figure S8: Optical microscope images (×10) of the resolution patterns for the acetone-based CuO/PMMA nanocomposite films. On the left hand-side the name of the samples is indicated, while the top row indicates the base dose value with proximity effect correction (in μC/cm^2^). Feature size (**a**) L_w_ = 300 nm, (**b**) L_w_ = 500 nm, (**c**) L_w_ = 1 μm, (**d**) L_w_ = 5 μm, (**e**) L_w_ = 10 μm and (**f**) L_w_ = 20 μm, Figure S9: Optical microscope images (×10) of the resolution patterns for the acetone-based CuO/PMMA nanocomposite films. On the left hand-side the name of the samples is indicated, while the top row indicates the base dose value with proximity effect correction (in μC/cm^2^). Feature size (**a**) L_w_ = 300 nm, (**b**) L_w_ = 500 nm, (**c**) L_w_ = 1 μm, (**d**) L_w_ = 5 μm, (**e**) L_w_ = 10 μm and (**f**) L_w_ = 20 μm, Table S1: Summary of CuO nanofiller synthesis conditions per sample.
