# Supplementary material for: CuO/PMMA Polymer Nanocomposites as Novel Resist Materials for E-Beam Lithography
Source: Nanomaterials (Basel). 2021 Mar 17;11(3):762. doi: 10.3390/nano11030762 (PMC8003112; doi:10.3390/nano11030762)
Supplement: Supplementary file 1 [file nanomaterials-11-00762-s001.pdf]

nanomaterials

CuO/PMMA Polymer Nanocomposites as Novel Resist Materials for E-Beam Lithography

Georgia Geka [1,2], George Papageorgiou [2], Margarita Chatzichristidi [1,*], Andreas Germanos Karydas [3], Vassilis Psycharis [2] and Eleni Makarona [2,*]

1. CuO Nanostructure Aqueous Synthesis

Throughout this work, wet chemical synthesis was employed for the production of CuO nanoparticles with a main target of producing in a cost-efficient and relatively simple manner well-defined, uniformly-sized nanostructures. Towards this target, the hydrolysis route of copper salts was opted and various combinations with regards to the salt concentration and the synthesis temperature were studied. The general route was the following and is depicted in Figure S1: Copper (II) acetate monohydrate is dissolved under constant magnetic stirring in 20 mL de-ionized water, so that the final solution has a concentration of 50 mM, 100 mM or 200 mM. The final solution is heated up to a pre-specified temperature (70 °C, 80 °C or 90 °C) under continuous magnetic stirring on a hot plate. An aqueous solution of NaOH 500 mM is added drop-wise (in 2mL doses) to the copper acetate solution until the molar ratio of NaOH to copper salt of 1:4. The blue-colored translucent solution gradually turns transparent as a black precipitate is formed, indicating the synthesis of CuO. The solution is left under constant stirring for 2 h at the final temperature. After the 2 h-interval the solution is left undisturbed to cool down to room temperature. The black precipitate is centrifuged and washed with de-ionized water three times. The final product is dried in oven for 21 h at 60 °C and 24 h at 90 °C.

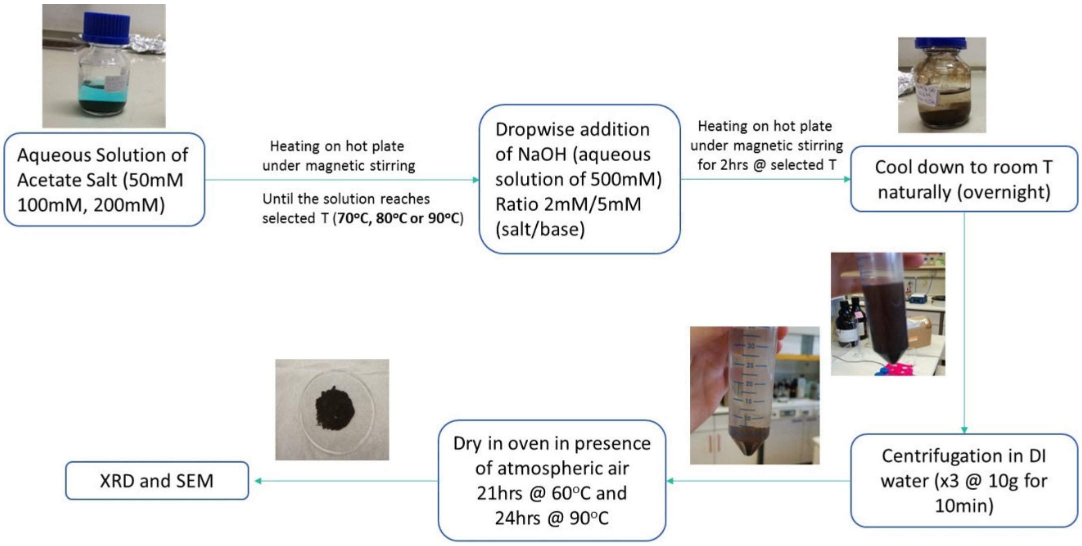

Figure S1. Schematic Representation of the CuO Nanofiller Synthesis.

The most pertinent combinations to this work are listed below in Table S1 and comprise the variation of the copper salt concentration (30 mM, 65 mM and 100 mM) and the synthesis temperature (70 °C, 80 °C and 90 °C).

Table 1. Summary of CuO nanofiller synthesis conditions per sample.

Sample Name	Copper (II) acetate monohydrate Concentration (mM)	Synthesis Temperature (°C)
CuO-30-70	30	70
CuO-30-80	30	80
CuO-30-90	30	90
CuO-65-70	65	70
CuO-65-80	65	80
CuO-65-90	65	90
CuO-100-70	100	70
CuO-100-80	100	80
CuO-100-90	100	90

The dried powders were characterized by X-ray diffraction (XRD) and Scanning Electron Microscopy (SEM) in order to elaborate the morphological and structural characteristics of the produced nanostructures. Representative SEM images are summarized in Figure S2, while the XRD spectra are compiled in Figure S3a. From the XRD data it can be readily seen that no matter the conditions of the synthesis, the produced nanostructures are of pure monoclinic phase of CuO. The diffraction peaks are sharp and no other impurity peaks were observed, indicating a high crystallinity and purity (Figure S3a). However, both the temperature and the copper salt concentration affect in a significant way the final nanostructure morphology (Figure S2) as well as the crystallite size as calculated from the XRD measurements. Based on the XRD diagrams, since the broadening of diffraction peaks is isotropic, the average size of CuO crystallites was determined from the FWHM of an isolated peak i.e. (−202), using the Scherrer equation and taking into account the instrumental broadening (Figure S3b).

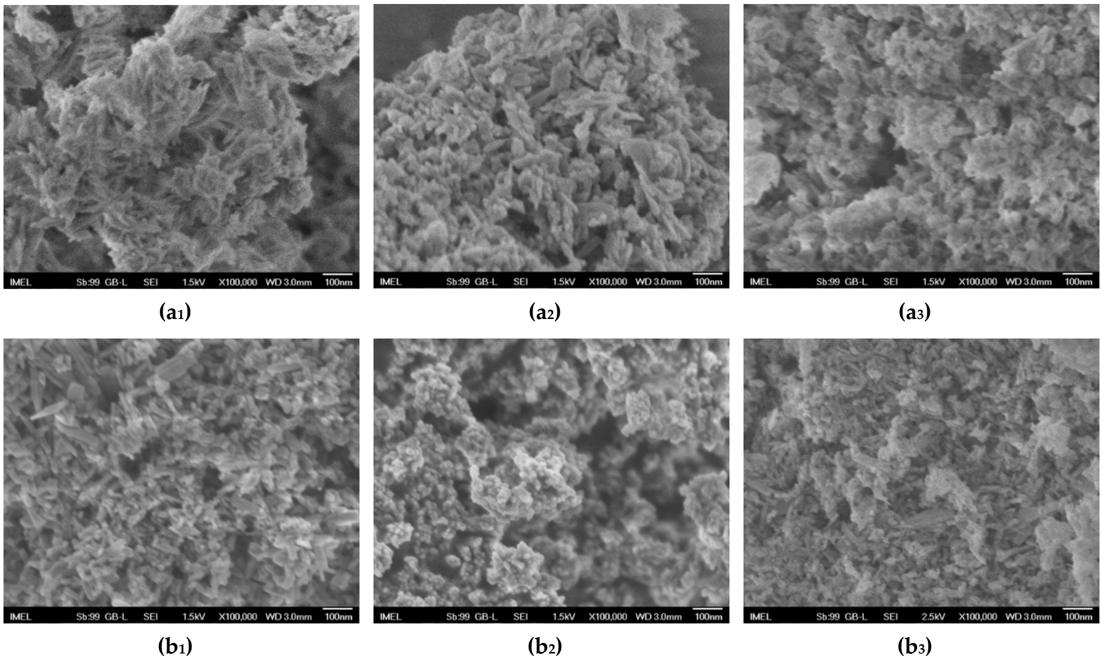

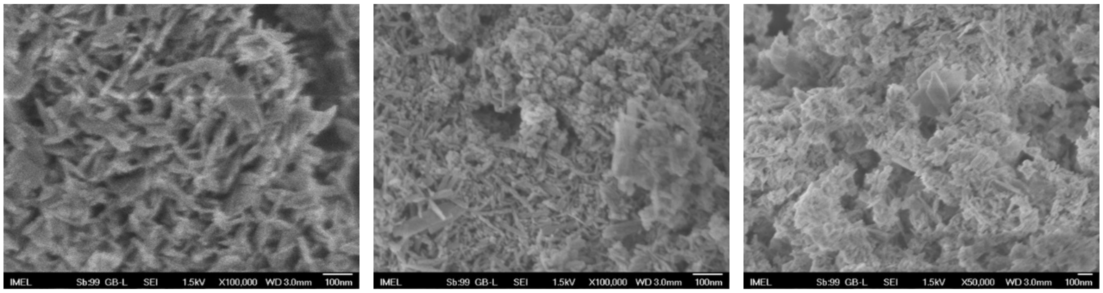

(c1) (c2) (c3)

Figure S2. SEM images of the nanopowders produced with copper (II) acetate monohydrate concentration of (**a**) 30 mM, (**b**) 65 mM and (**c**) 100 mM. Subscripts denote the temperature of synthesis: (1) 70 °C, (2) 80 °C and (3) 90 °C. Scale bar in all images: 100 nm ; magnification: x100,000.

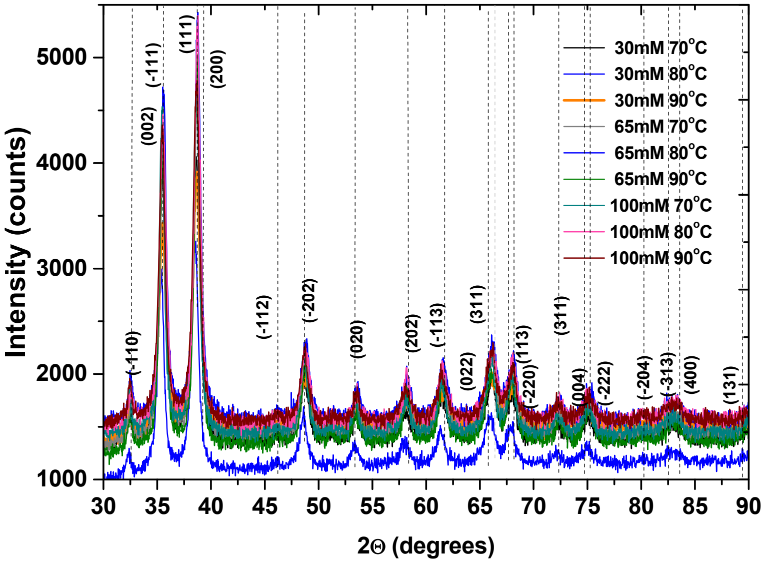

(a)

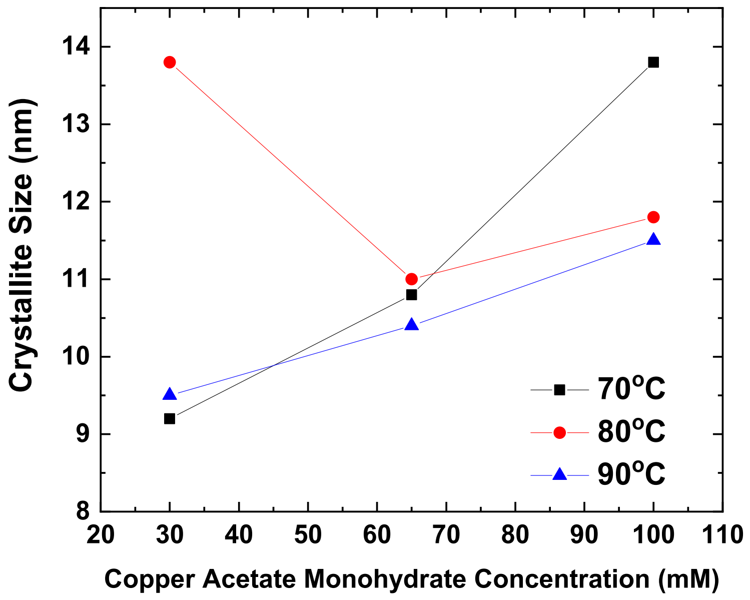

(b)

Figure S3. (**a**) XRD spectra of the nanopowders compared to the monoclinic phase of CuO (JCPDS pattern no 45-09370) demonstrating the pure monoclinic phase of all samples; (**b**) Crystallite size as function of copper (II) acetate monohydrate concentration for the three synthesis temperatures of 70 °C (black squares), 80 °C (red circles) and 90 °C (blue triangles).

2. EBL Patterning

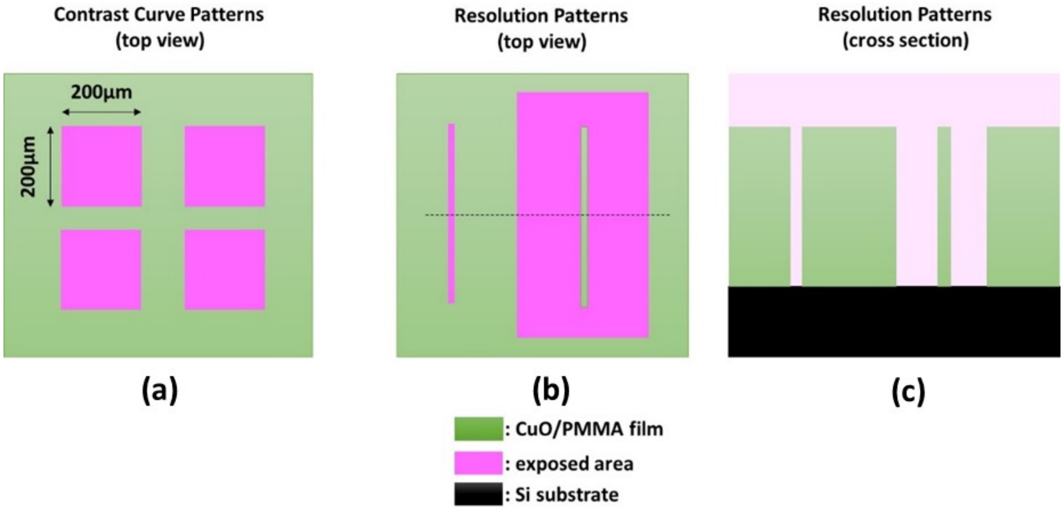

Figure S4. Schematics of (**a**) the contrast curve patterns (top view), (**b**) the resolution patterns (top view), and (**c**) the cross-section of the resolution patterns showing the wells and ridges.

3. CuO/PMMA Solution Stability

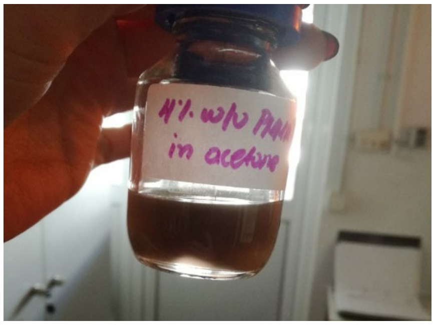

(a)

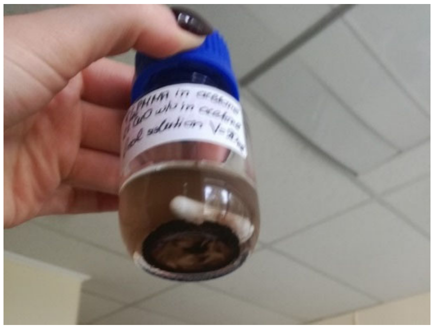

(b)

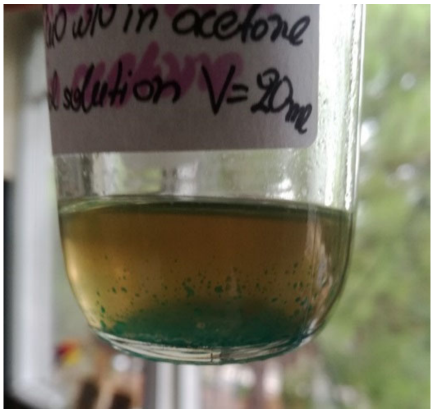

(c)

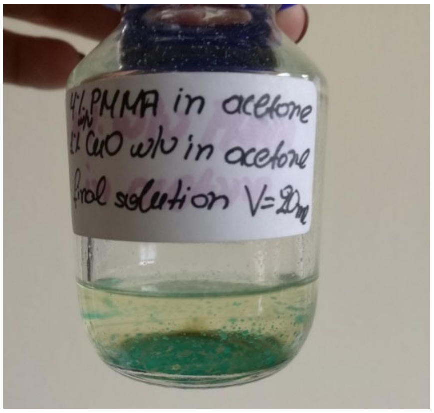

(d)

Figure S5. Photographs of the CuO/PMMA solutions (**a**) 1%CuO/PMMA after production and just prior to use for sample spin-coating, (**b**) 1%CuO/PMMA after 48 h stored in ambient conditions, (**c**) 1%CuO/PMMA with deflocculant after 2 weeks stored in ambient conditions, and (**d**) 1%CuO/PMMA with deflocculant after 1 month stored in ambient conditions.

4. Contrast and Resolution Patterns for Phase 2 Nanocomposite Films

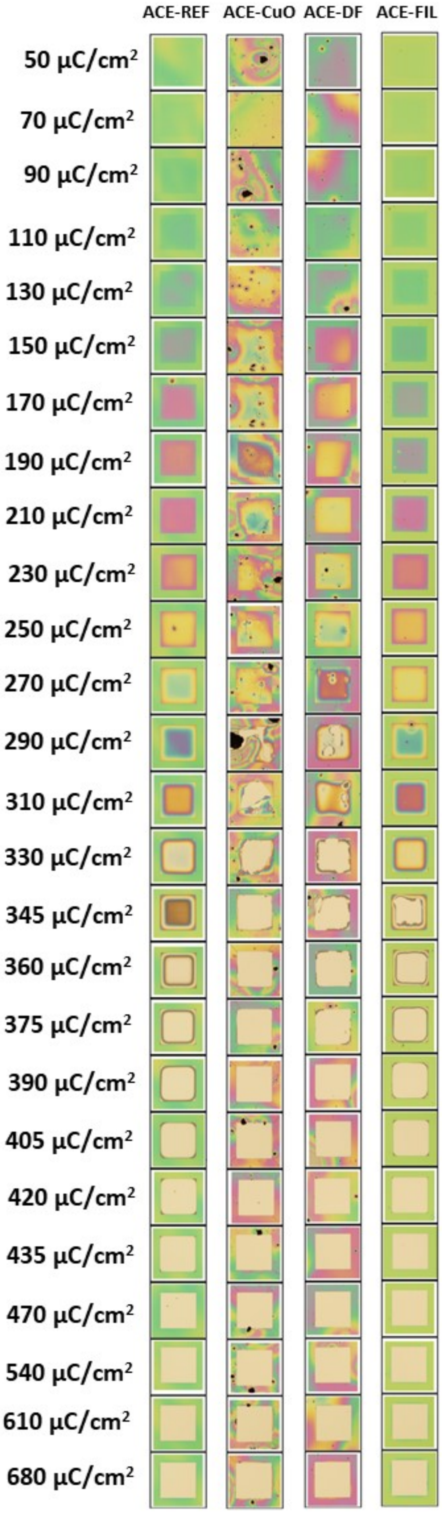

Figure S6. Optical microscope images (x10) of the contrast patterns for the acetone-based CuO/PMMA nanocomposite films.

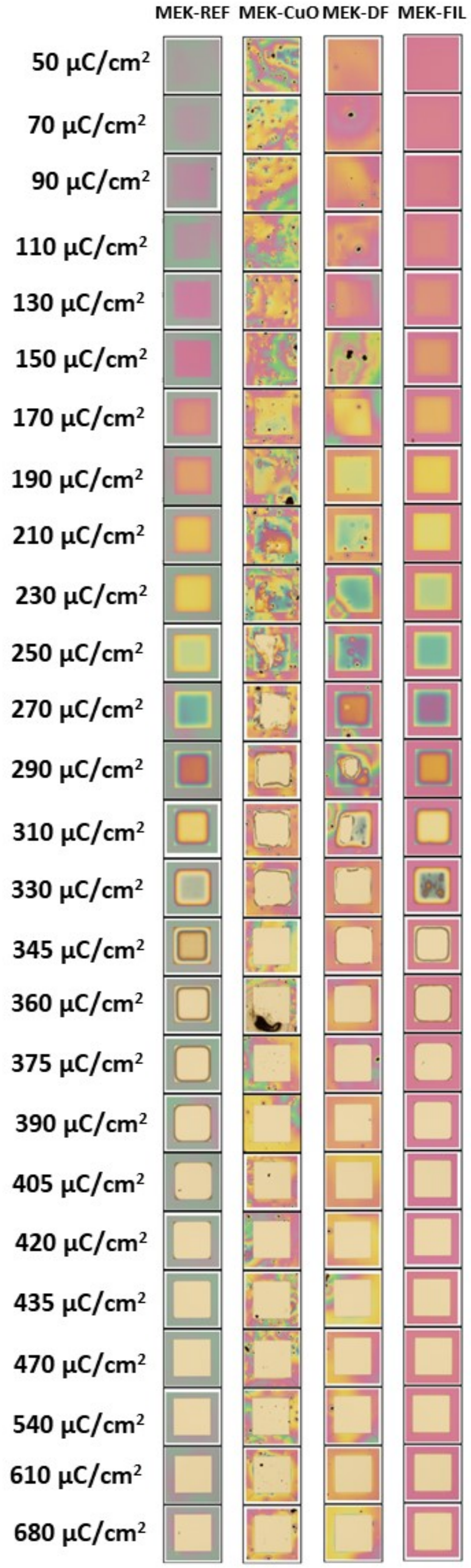

Figure S7. Optical microscope images (x10) of the contrast patterns for the MEK-based CuO/PMMA nanocomposite films. Base doses are indicated on the left-hand side.

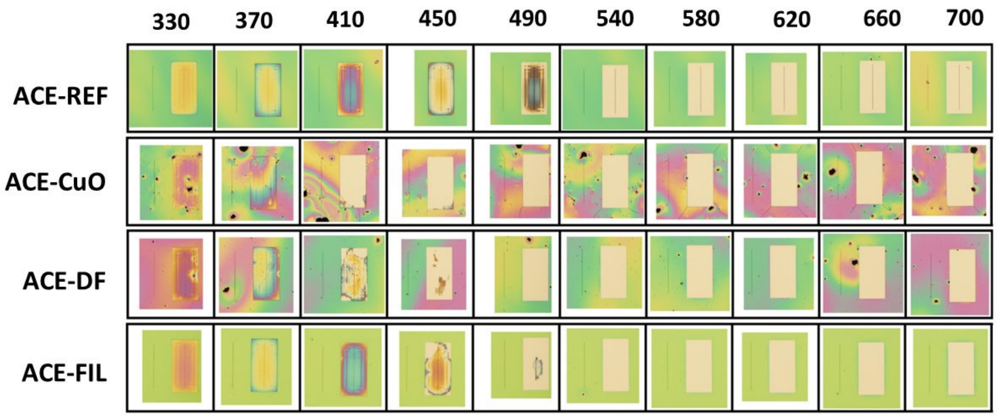
330
370
410
450
490
540
580
620
660
700
ACE-REF
ACE-CuO
ACE-DF
ACE-FIL

(a)

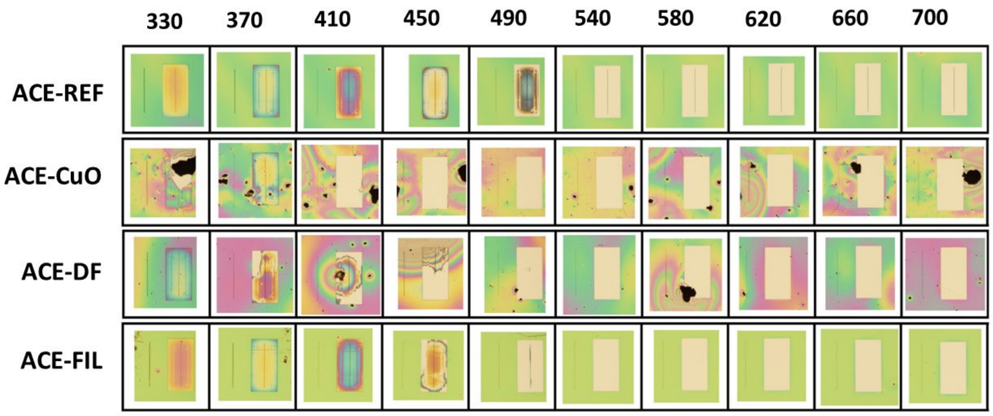
330
370
410
450
490
540
580
620
660
700
ACE-REF
ACE-CuO
ACE-DF
ACE-FIL

(b)

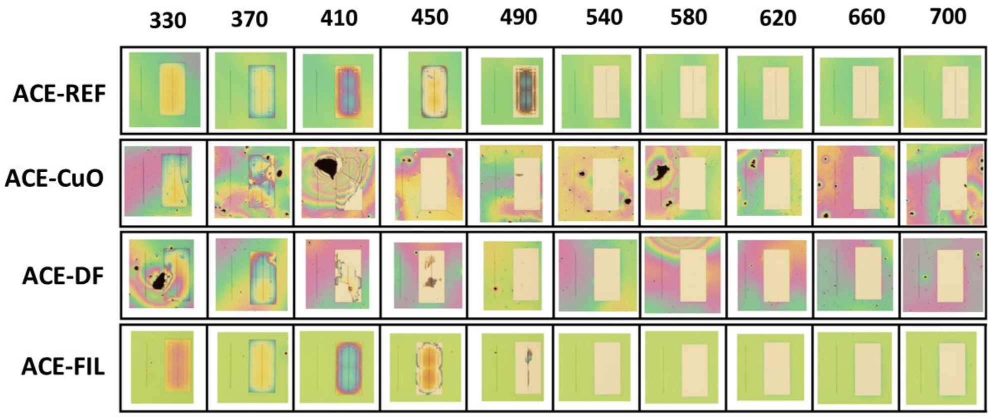
330
370
410
450
490
540
580
620
660
700
ACE-REF
ACE-CuO
ACE-DF
ACE-FIL

(c)

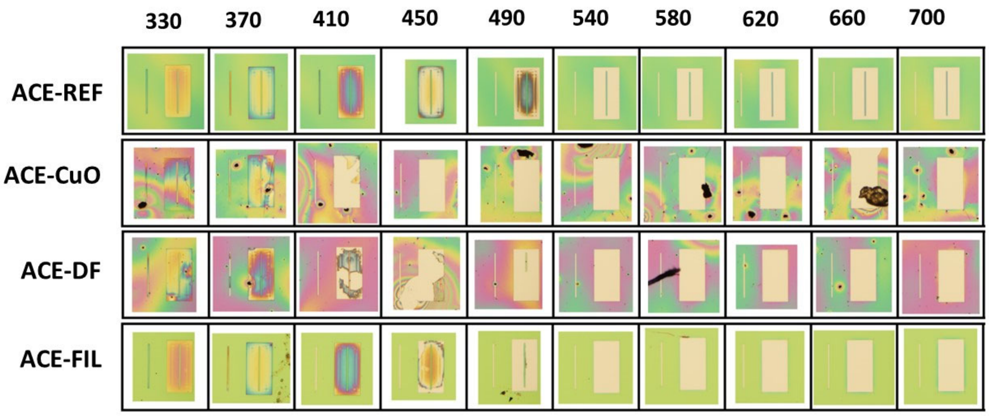

(d)

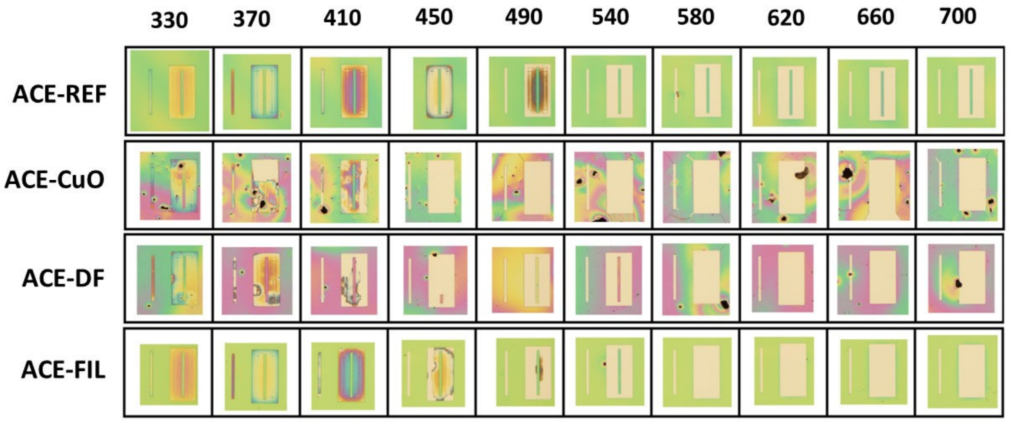

(e)

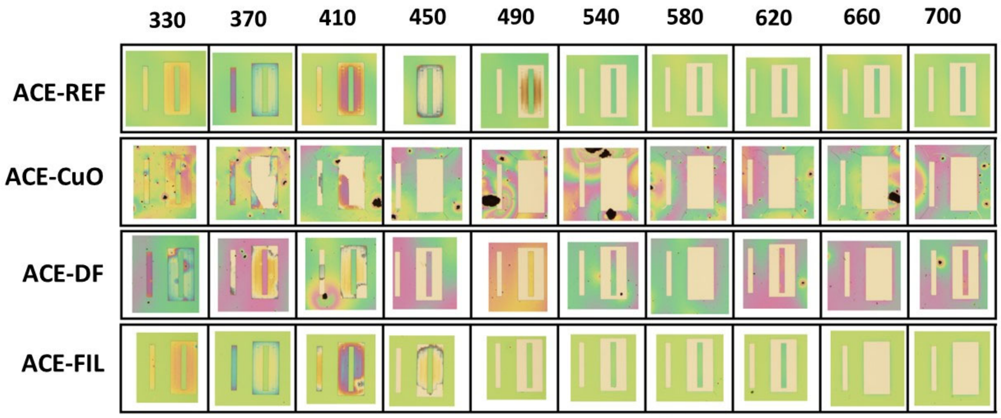

(f)

Figure S8. Optical microscope images (x10) of the resolution patterns for the acetone-based CuO/PMMA nanocomposite films. On the left hand-side the name of the samples is indicated, while the top row indicates the base dose value with proximity effect correction (in $\mu C/cm^2$). Feature size (**a**) L_w = 300 nm, (**b**) L_w = 500 nm, (**c**) L_w = 1 μm, (**d**) L_w = 5 μm, (**e**) L_w = 10 μm and (**f**) L_w = 20 μm.

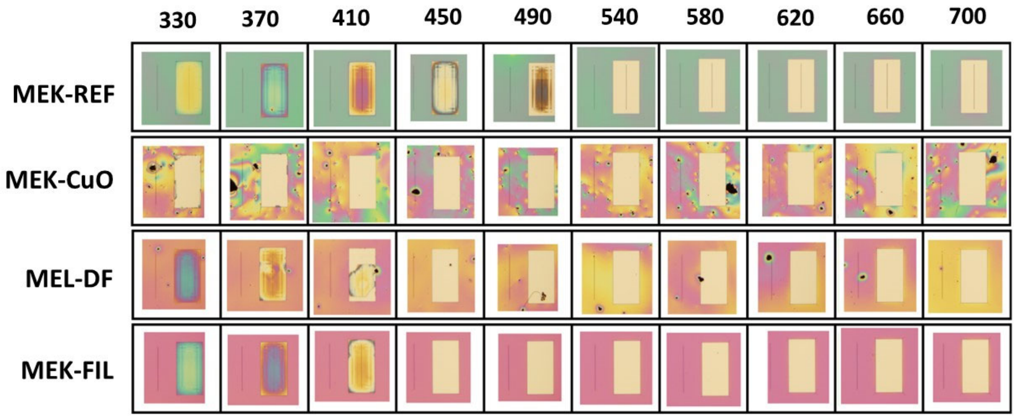
330
370
410
450
490
540
580
620
660
700
MEK-REF
MEK-CuO
MEL-DF
MEK-FIL

(a)

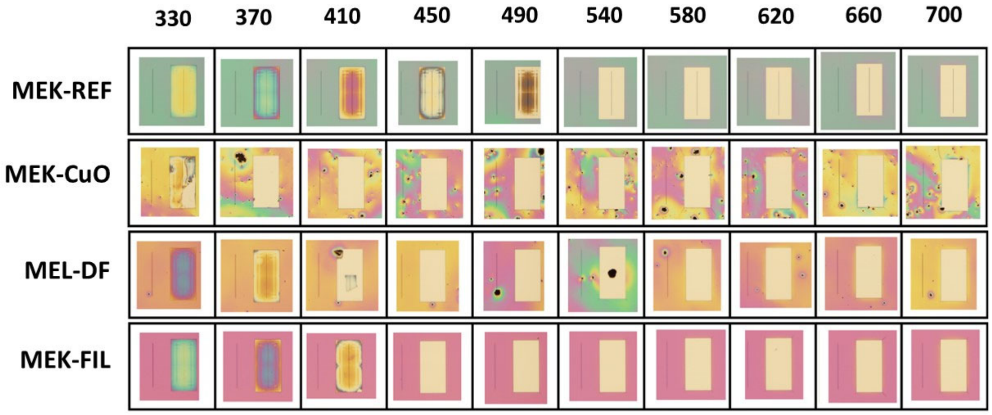
330
370
410
450
490
540
580
620
660
700
MEK-REF
MEK-CuO
MEL-DF
MEK-FIL

(b)

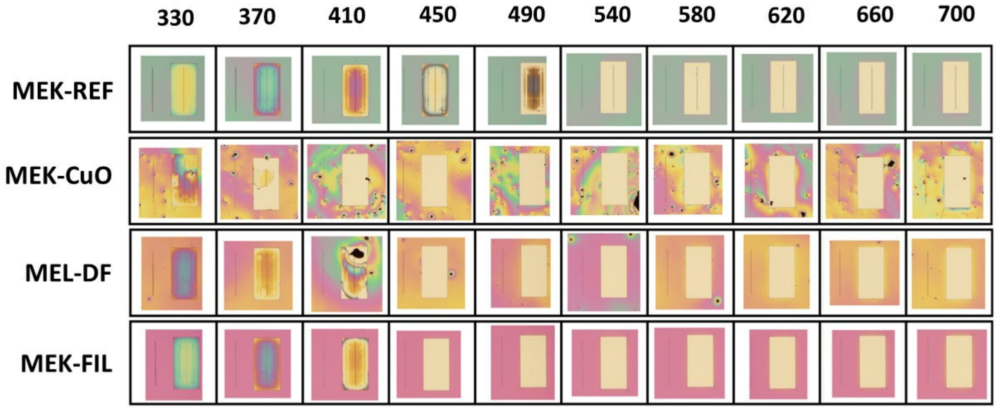
330
370
410
450
490
540
580
620
660
700
MEK-REF
MEK-CuO
MEL-DF
MEK-FIL

(c)

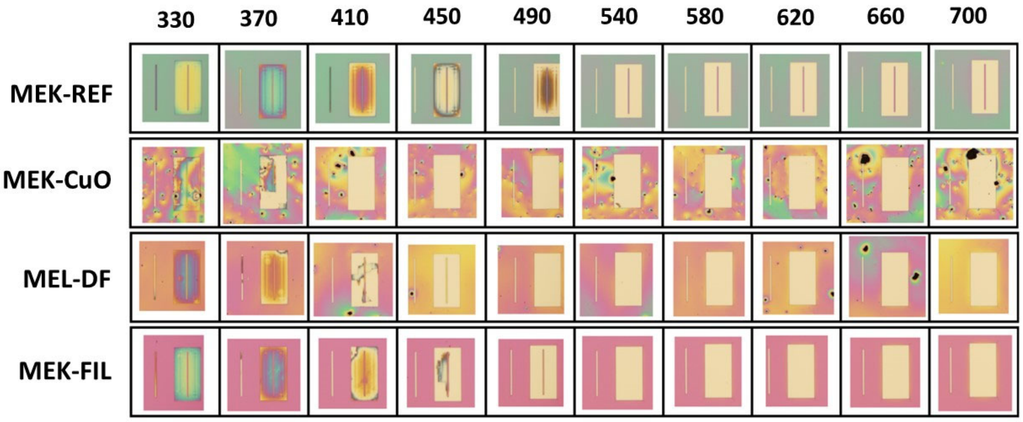

(d)

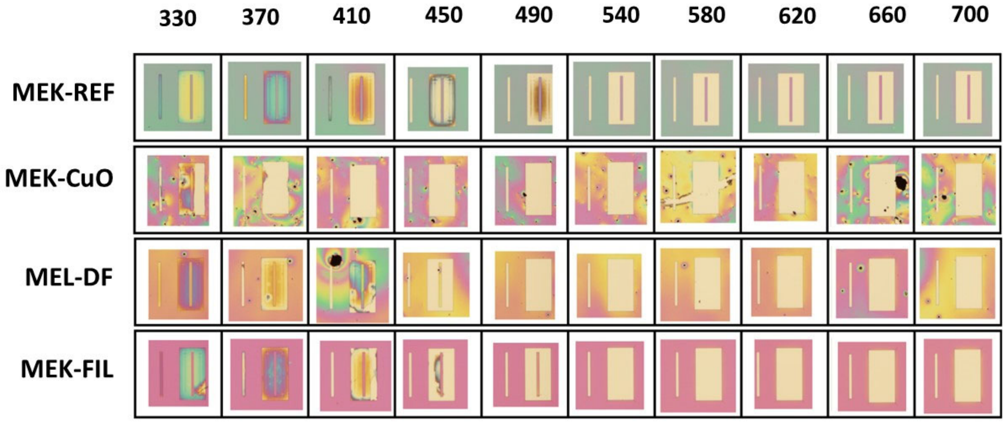

(e)

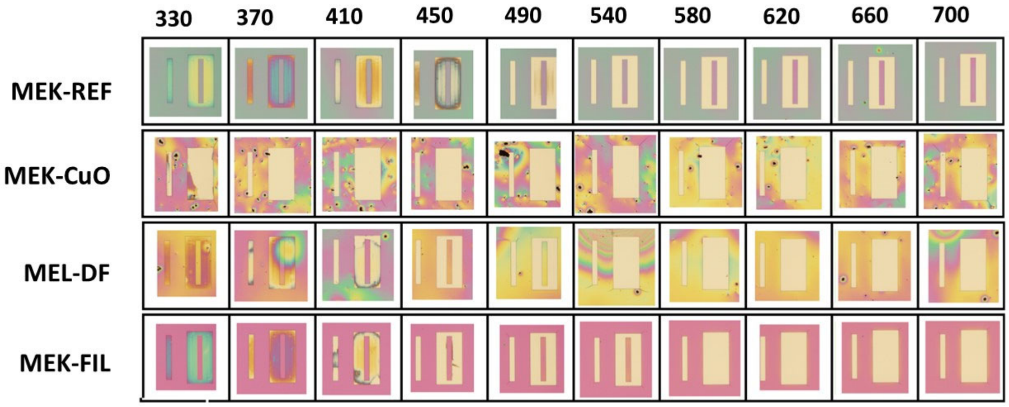

(f)

Figure S9. Optical microscope images (x10) of the resolution patterns for the MEK-based CuO/PMMA nanocomposite films. On the left hand-side the name of the samples is indicated, while the top row indicates the base dose value with proximity effect correction (in μC/cm²). Feature size (**a**) L_w = 300 nm, (**b**) L_w = 500 nm, (**c**) L_w = 1 μm, (**d**) L_w = 5 μm, (**e**) L_w = 10 μm and (**f**) L_w = 20 μm.
